# Effect of Nursing Intervention Supported by Mobile Health Applications on Asthma Control, Maternal and Neonatal Outcomes Among Pregnant Women with Asthma

**DOI:** 10.3390/healthcare14183079

**Published:** 2026-09-19

**Authors:** Hanan E. Nada, Ishraga Abdelgadir Ibrahim Mohamed, Faten Shawky Kandil, Hanan G. El-Bready, Fatma Ahmed Elsobkey, Safaa Gaber Aly Salem, Marwa A. Shahin, Enas Mohamed Lotfy

**Affiliations:** 1 Maternal & Newborn Health Nursing, Faculty of Nursing, Menoufia University, Shibin Al Kawm 32511, Egypt; hanan.alsayed56@nursing.menofia.edu.eg; 2 Critical Care Nursing, Department of Nursing, College of Applied Medical Sciences, University of Jeddah, Jeddah 21589, Saudi Arabia; imohamed@uj.edu.sa; 3 Department of Nursing, College of Applied Medical Sciences, University of Jeddah, Jeddah 21589, Saudi Arabia; fskandil@uj.edu.sa (F.S.K.); faelsobky@uj.edu.sa (F.A.E.); 4 Community Health Nursing, Faculty of Nursing, Menoufia University, Shibin Al Kawm 32511, Egypt; hanan.gamal@nursing.menofia.edu.eg; 5 Department of Pediatric Nursing, Faculty of Nursing, Benha University, Benha 13511, Egypt; 6 Department of Maternity, Obst & Gyn Nursing, College of Nursing, Princess Nourah bint Abdulrahman University, P.O. Box 84428, Riyadh 11671, Saudi Arabia; 7 Nursing Program, Batterjee Medical College, Jeddah 21442, Saudi Arabia; 8 Leadership and Management Nursing, Public Health Nursing Department, Faculty of Nursing, Northern Border University, Arar 73213, Saudi Arabia; enas.mahmoud@nbu.edu.sa

**Keywords:** nursing intervention, mobile health, Ailyn, asthma control, treatment adherence, self-monitoring, maternal outcomes, neonatal outcomes, pregnancy

## Abstract

Background: Asthma during pregnancy is associated with adverse maternal and neonatal outcomes. Nursing education, self-management support, and mobile health (mHealth) technologies may support asthma management during pregnancy. Aim: This study evaluated the effect of a structured nursing intervention supported by an mHealth application on asthma control, treatment adherence, and self-monitoring, and maternal and neonatal outcomes among pregnant women with asthma. Design and Setting: A quasi-experimental pretest–posttest design with a control group was conducted at two Maternal and Child Health Centers in Shebin El-Kom, Menoufia Governorate, Egypt. Participants: A convenience sample of 140 pregnant women with physician-diagnosed asthma was recruited and allocated non-randomly according to the center attended to a study group (*n* = 70) and a control group (*n* = 70). The sample size was calculated a priori based on a previous study using a 5% significance level and 80% statistical power, with a 1:1 allocation ratio, resulting in a required sample of 70 participants per group. All recruited participants completed the study, with no withdrawals or loss to follow-up. Intervention: The study group received routine antenatal care plus an eight-week structured nursing intervention consisting of four individualized weekly educational sessions (60–90 min each) addressing asthma management, medication adherence, self-monitoring, trigger avoidance, warning signs, inhaler technique, lifestyle modification, and rhythmic breathing exercises. Participants were trained to use the Airlyn mHealth application for guided breathing exercises, practiced 2–3 times daily, and received telephone/WhatsApp follow-up during weeks 6–8. The control group received routine antenatal care according to usual MCH center procedures. Measures: Asthma control was assessed using the Asthma Control Test (ACT). Treatment adherence and self-monitoring were assessed using the Asthma Adherence and Self-Monitoring Questionnaire. Maternal outcomes included hypertensive disorders, gestational diabetes, infections, preterm labor/PPROM, hemorrhage, and mode of delivery. Neonatal outcomes included birth weight, Apgar scores, respiratory distress and respiratory support, NICU admission and length of stay, and condition at discharge. The researcher-developed instruments underwent expert assessment of content validity and assessment of internal consistency before use. Results: Baseline ACT scores were comparable between the study and control groups (14.5 ± 4.2 vs. 14.1 ± 4.3; mean difference = 0.4; *p* = 0.580). Following the intervention, ACT scores were higher in the study group than in the control group (19.7 ± 3.6 vs. 15.2 ± 4.2; mean difference = 4.5; 95% CI: 3.189–5.811; *p* < 0.001; Cohen’s d = 1.147). Well-controlled asthma was observed in 75.7% of the study group compared with 17.1% of the control group (*p* < 0.001). Treatment adherence and self-monitoring scores were also better in the study group (5.6 ± 2.7 vs. 9.1 ± 3.1; mean difference = −3.5; 95% CI: −4.476 to −2.524; *p* < 0.001; Cohen’s d = 1.199). The overall maternal outcome score was more favorable in the study group (3.5 ± 1.7 vs. 6.1 ± 2.6; mean difference = −2.6; 95% CI: −3.340 to −1.860; *p* < 0.001; Cohen’s d = 1.174), as was the overall neonatal outcome score (7.7 ± 2.2 vs. 5.5 ± 2.1; mean difference = 2.2; 95% CI: 1.480–2.920; *p* < 0.001; Cohen’s d = 1.022). Significant between-group differences were also observed in birth weight, Apgar scores, respiratory distress, and NICU admission. Conclusions: Participants who received the structured nursing intervention supported by the Airlyn mHealth application were associated with improved asthma control, treatment adherence, and self-monitoring, as well as more favorable maternal and neonatal outcomes, compared with those receiving routine antenatal care. However, the quasi-experimental design, convenience sampling, and non-randomized allocation limit causal interpretation and the generalizability of the findings. Therefore, the findings should be interpreted cautiously. Recommendations: Larger randomized controlled trials with standardized protocols, adequate sample sizes to evaluate maternal and neonatal outcomes, and longer follow-up periods are recommended to confirm these findings and further assess their clinical relevance.

## 1. Introduction

Asthma is a chronic inflammatory disorder of the airways characterized by variable airflow limitation and recurrent episodes of cough, wheezing, chest tightness, and shortness of breath. Affecting approximately 4–12% of women of reproductive age, asthma represents the most common chronic respiratory condition complicating pregnancy and is primarily managed within primary healthcare settings [1].

Pregnancy involves marked physiological, hormonal, and immunological changes that can influence asthma control. Variations in estrogen and progesterone levels may affect airway mucosal edema, mucus secretion, airway smooth muscle tone, and bronchial responsiveness, whereas pregnancy-induced immunological adaptations may alter inflammatory processes [1,2]. In addition, cardiopulmonary changes during pregnancy, including increased oxygen demand, greater tidal volume and minute ventilation, elevation of the diaphragm, and changes in lung volumes during the later stages of pregnancy, may further affect respiratory function and contribute to changes in asthma status throughout gestation [1,2]. Consequently, asthma may improve, remain unchanged, or deteriorate during pregnancy, with symptom worsening occurring in a substantial proportion of pregnant women, particularly those with more severe disease [1,2,3].

Despite considerable advances in pharmacological therapy, poorly controlled asthma during pregnancy continues to be an important cause of maternal and neonatal morbidity [2,4,5,6]. Maternal asthma has been associated with an increased likelihood of several pregnancy-related complications, including preeclampsia, placental disorders, obstetric hemorrhage, spontaneous abortion, gestational diabetes, pulmonary embolism, preterm birth, and cesarean delivery [2,5]. In addition, maternal asthma may adversely affect fetal and neonatal health. Infants born to mothers with asthma have been reported to have increased risks of low birth weight, small-for-gestational-age status, preterm birth, congenital anomalies, neonatal hospitalization, and perinatal mortality [3,4,5]. Therefore, achieving adequate asthma control during pregnancy is essential not only for maternal health but also for fetal and neonatal well-being.

Substantial evidence has demonstrated associations between maternal asthma and adverse perinatal outcomes. Murphy et al. (2011) [4], in a systematic review and meta-analysis, reported significant associations between maternal asthma and adverse perinatal outcomes, including low birth weight, preterm birth, and small-for-gestational-age infants. The review also identified increased risks of congenital malformations, neonatal death, and hospitalization. Similarly, Mendola et al. (2014) [5] reported adverse neonatal health outcomes among infants born to mothers with asthma. More recently, Robijn et al. (2024) [6], in an updated systematic review and meta-analysis, further examined adverse neonatal outcomes associated with asthma during pregnancy. Collectively, these findings highlight the importance of effective asthma management during pregnancy to minimize potential adverse maternal and neonatal consequences [4,5,6].

The mechanisms through which maternal asthma may contribute to adverse fetal and neonatal outcomes have not been fully clarified. Proposed mechanisms include maternal hypoxia, which may impair fetal oxygenation and placental function, as well as asthma-related inflammatory processes that may affect placental and fetal development [1,2,5]. In addition, concerns about medication use during pregnancy and inadequate adherence to prescribed treatment may contribute to poor asthma control and an increased risk of exacerbations [1,2,5]. Consequently, effective asthma management during pregnancy requires not only appropriate pharmacological treatment but also education, adherence support, symptom monitoring, and effective self-management strategies.

Effective asthma self-management is an essential component of optimal asthma care during pregnancy. However, evidence indicates that pregnant women may experience insufficient knowledge of asthma management, inadequate self-management skills, and difficulties with adherence to prescribed controller medications [7]. Zairina et al. (2014) [7], in a systematic review of non-pharmacological healthcare interventions for asthma management during pregnancy, highlighted the limited evidence available for such interventions and emphasized the need for well-designed studies involving pregnant women. Asthma control may also vary considerably throughout pregnancy. Schatz et al. (1988) [3], in a prospective analysis of pregnancies among women with asthma, demonstrated that the course of asthma during pregnancy was variable, with some women experiencing improvement and others experiencing deterioration or no significant change. These findings emphasize the importance of continuous assessment and individualized support throughout pregnancy.

Nurses play an important role in supporting asthma management during pregnancy through patient education, reinforcement of medication adherence, symptom assessment, promotion of self-care, and communication with healthcare providers. Nursing interventions may include education regarding asthma triggers, appropriate medication and inhaler use, breathing exercises, symptom recognition, and appropriate responses to worsening asthma. Murphy et al. (2005) [8] demonstrated the potential value of structured asthma education and self-management support during pregnancy, including improvements in asthma knowledge, inhaler technique, medication adherence, and self-management skills. Therefore, nurse-led interventions may represent an important strategy for improving asthma control, treatment adherence, and self-management among pregnant women with asthma [7,8].

Recent advances in digital health technologies have provided new opportunities to strengthen chronic disease management and extend healthcare support beyond routine clinical encounters. Mobile health (mHealth) interventions use mobile devices and applications to provide health education, monitoring, treatment support, communication, and self-management assistance. Evidence from pregnancy-related mHealth research has demonstrated the potential of mobile interventions to support health-related behaviors and disease management among pregnant women [9]. In asthma care, mobile applications may provide medication reminders, symptom monitoring, educational materials, asthma self-management support, and communication with healthcare providers [10,11,12,13]. These functions may complement conventional asthma care by allowing patients to access information and support between healthcare visits.

Treatment adherence is particularly important in chronic asthma management because inconsistent use of prescribed therapy may compromise asthma control. mHealth applications may provide practical support for adherence through medication reminders, repeated educational messages, symptom monitoring, and reinforcement of self-management behaviors. Mohamed et al. (2025) [10] investigated the efficacy of a phone application in relation to treatment adherence among patients with asthma, supporting the potential relevance of mobile-based approaches to asthma management. However, the applicability of such interventions to pregnant women with asthma remains uncertain because pregnancy represents a distinct clinical context requiring individualized monitoring and support.

Recent evidence also indicates that the effectiveness of digital asthma interventions may vary according to intervention characteristics and patient populations. A systematic review of digital interventions for asthma reported potential benefits for medication adherence and asthma control, although the certainty of evidence varied across outcomes and intervention types [11]. Schulte et al. (2021) [12], in a systematic review of eHealth interventions for asthma and chronic obstructive pulmonary disease, reported variable effects on medication adherence among patients with asthma. Nguyen et al. (2021) [13] also reviewed mHealth applications interfacing with inhaler sensors and highlighted their potential to support asthma self-management while identifying limitations in the available evidence for some clinically important outcomes. These findings suggest that although digital technologies have considerable potential, their effectiveness may depend on the intervention design, level of patient support, and integration of technology into comprehensive care [11,12,13].

Despite the growing evidence regarding digital health and asthma self-management, evidence specifically addressing pregnant women with asthma remains limited. Previous pregnancy-related research has largely focused on pharmacological management and non-pharmacological asthma education, whereas digital approaches have predominantly been evaluated in broader asthma populations [7,8,11,12,13]. Furthermore, previous studies have generally examined nursing education, non-pharmacological interventions, or digital health strategies separately. Limited evidence is available regarding an integrated nursing intervention supported by an mHealth application specifically designed for pregnant women with asthma. In particular, the potential effects of such an integrated intervention on asthma control, treatment adherence, self-monitoring, and maternal and neonatal outcomes have not been sufficiently addressed.

This gap is clinically relevant because routine antenatal visits may provide limited opportunities for repeated education, adherence reinforcement, symptom monitoring, and self-management support. Integrating nursing interventions with mHealth technology may extend support beyond the clinical setting and provide pregnant women with continuous access to educational content, reminders, symptom-monitoring guidance, and self-management support. Such integration may therefore address some of the limitations of conventional education-based approaches and strengthen continuity of asthma care during pregnancy [7,10,11,12,13].

The novelty of the present study lies in integrating a structured nursing intervention with a mobile health application specifically for pregnant women with asthma. Unlike previous studies that have primarily examined asthma education, non-pharmacological interventions, or digital health strategies separately, the present study combines nursing education, self-management support, adherence reinforcement, symptom monitoring, and mobile-based support within a single intervention. Furthermore, the study evaluates multiple outcomes, including asthma control, adherence, self-monitoring, and maternal and neonatal outcomes, providing a broader assessment of the potential clinical value of the intervention.

The main findings of the present study demonstrated highly statistically significant improvements in asthma control, adherence, and self-monitoring among pregnant women who received the nursing intervention supported by the mHealth application compared with those in the control group (*p* < 0.001). In addition, the intervention group achieved significantly better maternal and neonatal outcome scores than the control group (*p* < 0.001). These findings provide preliminary evidence that integrating structured nursing support with mHealth technology may be a valuable approach to strengthening asthma management and improving maternal and neonatal outcomes during pregnancy.

Accordingly, the present study was undertaken to evaluate the effect of a nursing intervention supported by a mobile health application on asthma control and maternal and neonatal outcomes among pregnant women with asthma.

### 1.1. Related Works

Previous studies have investigated different approaches to improving asthma management, including self-management education, nursing support, and digital health interventions. However, their populations, intervention components, and outcome measures have varied considerably.

Among pregnant women, Yoo et al. (2021) [14] evaluated an integrated asthma intervention that included assessment, education, and monitoring and reported improvement in asthma control among participating pregnant women. However, the study did not include a separate control group, limiting the ability to determine whether the observed improvement was attributable to the intervention alone. This highlights the need for controlled studies evaluating structured interventions specifically during pregnancy.

Evidence from broader asthma populations also supports the role of self-management and digital interventions. Dhippayom et al. (2022) [15] found that different forms of behavioral and self-management support could improve asthma-related outcomes, while Mosnaim et al. (2021) [16] reported that interactive digital interventions involving patient communication showed potential for improving selected asthma-management outcomes. Similarly, Doshi et al. (2021) [17], Nguyen et al. (2021) [13], Schulte et al. (2021) [12], and Chan et al. (2022) [11] demonstrated the potential value of technology-based approaches for supporting asthma management and medication adherence. Nevertheless, most of these studies were conducted in general asthma populations rather than specifically among pregnant women.

More recent evidence has examined smartphone-based and virtual approaches. Farzandipour et al. (2024) [18] evaluated a smartphone-based self-management intervention in patients with asthma and reported favorable effects on asthma control and quality of life. However, the study involved a relatively small non-pregnant population and did not assess maternal or neonatal outcomes. In contrast, Thomas et al. (2025) [19] evaluated virtual antenatal asthma care among pregnant women and demonstrated greater engagement with virtual care, although asthma control improved in both groups without a significant difference between them. Thus, the findings support the feasibility of digital approaches in antenatal asthma care but do not establish the superiority of a specific mHealth-supported nursing intervention.

Overall, previous research provides evidence supporting asthma education, self-management, and digital health approaches; however, pregnancy-specific evidence remains limited, particularly for integrated interventions combining structured nursing care with mHealth technology. Moreover, previous studies have generally focused on individual outcomes such as asthma control, medication adherence, or engagement, with limited evaluation of asthma control, adherence, self-monitoring, and maternal and neonatal outcomes together. Therefore, the present study was designed to address this gap by evaluating the effect of a structured nursing intervention supported by a mobile health application among pregnant women with asthma (Table 1).

### 1.2. Research Gap

Despite the growing evidence supporting self-management and digital health interventions in asthma care, there remains limited pregnancy-specific evidence evaluating an integrated nursing intervention supported by an mHealth application. Previous studies have generally examined asthma control, medication adherence, digital engagement, or self-management separately, while few have simultaneously assessed asthma control, adherence, self-monitoring, and maternal and neonatal outcomes. Therefore, the present study addresses an important gap by evaluating a structured nursing intervention supported by mHealth technology among pregnant women with asthma.

### 1.3. Research Hypotheses

1. Pregnant women with asthma who receive a nursing intervention supported by mobile health (mHealth) applications will demonstrate significantly better asthma control than those receiving routine antenatal care.
2. Pregnant women with asthma who receive a nursing intervention supported by mobile health (mHealth) applications will experience significantly better maternal outcomes than those receiving routine antenatal care.
3. Pregnant women with asthma who receive a nursing intervention supported by mobile health (mHealth) applications will experience significantly better neonatal outcomes than those receiving routine antenatal care.

### 1.4. Aim of the Study

To investigate the effect of a nursing intervention supported by a mobile health (mHealth) application on asthma control, maternal and neonatal outcomes among pregnant women with asthma.

## 2. Methods

### 2.1. Methodological Overview

This study investigated the effect of a structured nursing intervention supported by the Airlyn mobile health (mHealth) application on asthma-related outcomes among pregnant women with asthma. A total of 140 eligible participants were included and allocated equally to a study group and a control group according to the Maternal and Child Health (MCH) center they attended. The study group received an eight-week nursing intervention comprising structured educational sessions, asthma self-management support, and guided rhythmic breathing exercises supported by the Airlyn application, whereas the control group received routine antenatal care. Asthma control, treatment adherence and self-monitoring, maternal outcomes, and neonatal outcomes were assessed to evaluate the effect of the intervention.

### 2.2. Study Design

A quasi-experimental pretest–posttest design with a control group was employed to evaluate the effect of a structured nursing intervention supported by the Airlyn mobile health (mHealth) application on asthma control, treatment adherence and self-monitoring, and maternal and neonatal outcomes among pregnant women with asthma.

### 2.3. Study Setting

The study was conducted at two Maternal and Child Health Centers (Qebly and Bahary) in Shebin El-Kom, Menoufia Governorate, Egypt. The centers were selected because of the high flow of pregnant women from surrounding cities and villages, which facilitated recruitment of the required sample. Both centers provide comprehensive maternal and child health services, including antenatal, natal, postnatal, and family planning services. The antenatal clinics operate twice weekly, with an average attendance of 25–35 pregnant women per day.

### 2.4. Participants and Sampling

#### 2.4.1. Sampling Technique and Sample Size

A convenience sampling technique was used to recruit participants for the study. A total of 140 pregnant women who met the study’s inclusion criteria were recruited and included in the study.

In accordance with the data from the literature (Gebresilassie et al., 2025) [20], considering a level of significance of 5% and a power of 80%, the sample size was calculated using the following formula:

$$n\;=\;\frac{2(Z\alpha /2+Z\beta )2\times p(1-p)}{\left(d\right)2}$$

where p = pooled proportion obtained from a previous study, d = expected difference in proportion of events, Z_α/2_ = 1.96 (for 5% level of significance), and Z_β_ = 0.84 (for 80% power of the study). Therefore,

$$n=\frac{2{\left(1.96+0.84\right)}^{2}\times 0.469(1-0.469)}{\left(0.237\right)2}\;=\;69.5.$$

Accordingly, the required sample size was 70 participants per group. All recruited participants completed the study, with no withdrawals or loss to follow-up. Complete data on all maternal and neonatal outcomes were available for all 140 participants. Therefore, the final sample analyzed consisted of 140 participants, with 70 participants in the study group and 70 participants in the control group.

Given the use of convenience sampling within a quasi-experimental design and the non-randomized allocation of participants according to the Maternal and Child Health (MCH) Center attended, baseline comparability was specifically assessed before initiation of the intervention. Sociodemographic, obstetric, and asthma-related characteristics were measured using the same standardized assessment procedures and tools in both groups and were statistically compared to identify potential pre-existing differences that could influence the study outcomes.

#### 2.4.2. Inclusion and Exclusion Criteria

The inclusion criteria for this study included pregnant women between 12 and 28 weeks of gestation with a singleton pregnancy and confirmed diagnosis of asthma by a physician, irrespective of disease severity. willing to participate in the study and owned a mobile phone with the ability to use mobile health (mHealth) applications required for the intervention.

The exclusion criteria included women with multiple pregnancies, those presenting with major obstetric complications at the time of enrollment, such as severe preeclampsia or placenta previa, and women with chronic medical conditions that may independently influence maternal or neonatal outcomes, including cardiac or renal diseases.

### 2.5. Data Collection Instruments

Five main instruments were employed to collect the required data:

#### 2.5.1. Instrument I: A Structured Interviewing Questionnaire

It was developed by the researchers after reviewing the relevant literature to collect baseline information about the study participants. The instrument consisted of three parts. Part I assessed participants’ sociodemographic characteristics, including age, educational level, residence, and occupation. Part II collected participants’ obstetric history, including gravidity, parity, history and number of previous abortions, and gestational age of the current pregnancy. Part III: to assessed participants’ asthma-related characteristics. It included data on asthma duration, common asthma triggers, history of asthma-related hospitalization and emergency department visits, current asthma medications, treatment adherence (regular or irregular use), and physician-diagnosed asthma severity (mild, moderate, or severe). The questionnaire was administered by the researchers through face-to-face interviews using the same standardized procedure for all participants.

#### 2.5.2. Instrument II

The Asthma Control Test (ACT) is a standardized and validated questionnaire adopted from the Ministry of Health, Kingdom of Saudi Arabia (2025) [21] and used to assess asthma control. The ACT consists of five items that evaluate asthma control during the previous four weeks, including activity limitation due to asthma, frequency of shortness of breath, nocturnal symptoms, use of rescue medication, and the participant’s overall rating of asthma control. Each item is rated on a 5-point Likert scale, with response options ranging from 1 to 5, resulting in a total score ranging from 5 to 25, with higher scores indicating better asthma control. The ACT was administered by the researchers through face-to-face interviews using a standardized procedure for all participants.

Scoring system: The total score was calculated by summing the scores of the five items, yielding a possible range of 5–25. According to the recommended interpretation, a total score of ≤19 indicated uncontrolled asthma, whereas a score of 20–25 indicated well-controlled asthma.

#### 2.5.3. Tool IV: Asthma Adherence and Self-Monitoring Questionnaire

The Asthma Adherence and Self-Monitoring Questionnaire was developed by the researcher following an extensive review of the relevant literature and international clinical guidelines, particularly the Global Initiative for Asthma (GINA) guidelines (2025), as well as the studies by Miller et al. (2017) and Hui et al. (2017) [22,23,24]. The reviewed literature and guidelines were used to identify relevant domains related to asthma symptoms, treatment adherence, and self-monitoring. Based on these domains, the researcher developed seven items addressing recent daytime asthma symptoms, nighttime symptoms, nocturnal awakening due to asthma, activity limitation due to asthma, use of reliever medication, adherence to controller medication, and emergency healthcare visits due to asthma.

Participants completed the questionnaire once at baseline and once at the post-intervention assessment. They were asked to report their asthma-related symptoms, medication use, adherence to controller medication, self-monitoring behaviors, and asthma-related healthcare utilization during the preceding four weeks. The four-week recall period was selected to provide a broader assessment of recent asthma symptoms, treatment adherence, and self-monitoring behaviors while minimizing the potential recall bias associated with substantially longer recall periods. The questionnaire was administered at baseline and at the post-intervention assessment by the researchers to evaluate changes following the nursing intervention.

Scoring system: Each item was coded using an ordinal scoring system to reflect the severity or frequency of asthma symptoms, medication adherence, self-monitoring, and asthma-related healthcare utilization. Higher scores indicated poorer asthma adherence and self-monitoring. For daytime symptoms, nighttime symptoms, and activity limitation, responses were scored as follows: 0 = none, 1 = mild, 2 = moderate, and 3 = severe. Night awakening was scored as 0 = no awakening, 1 = once, and 2 = two or more times. Use of reliever medication (SABA) was scored as 0 = not used, 1 = used once, and 2 = used two or more times. Adherence to controller medication (ICS) was scored as 0 = adherent and 1 = non-adherent, while emergency visits due to asthma were scored as 0 = no and 1 = yes.

The total score ranged from 0 to 15. For descriptive purposes, the total score was categorized into three levels: 0–5 = good asthma adherence and self-monitoring, 6–10 = moderate asthma adherence and self-monitoring, and 11–15 = poor asthma adherence and self-monitoring. These categories were developed for the purposes of interpretation in the present study and were not considered validated clinical cut-off values.

#### 2.5.4. Instrument IV: Maternal Outcome Assessment Checklist

The Maternal Outcome Assessment Checklist was developed by the researchers based on current clinical guidelines and relevant literature concerning maternal outcomes associated with asthma during pregnancy, particularly the study by Li et al. (2024) [25]. The reviewed literature was used to identify maternal gestational and delivery-related outcomes relevant to the study population. These identified outcomes were organized into a structured checklist to facilitate standardized assessment of maternal outcomes.

The checklist consisted of nine items covering gestational hypertensive disorders, gestational diabetes mellitus, respiratory infections during pregnancy, urinary tract infection, preterm labor or preterm premature rupture of membranes (PPROM), antepartum hemorrhage, postpartum hemorrhage, other maternal complications, and mode of delivery. The checklist was completed by the researcher using the participants’ medical and obstetric records and relevant clinical information documented during pregnancy and delivery. The researcher reviewed the available records and recorded the presence or absence of each maternal complication and the mode of delivery using the predefined categories. This standardized procedure was used to ensure consistency in the assessment of maternal outcomes.

Scoring system: Each maternal complication was scored as 0 = no and 1 = yes. Mode of delivery was scored as 0 = vaginal delivery, 1 = elective cesarean section, and 2 = emergency cesarean section. The total score therefore ranged from 0 to 10. Higher scores indicated a greater occurrence of adverse maternal outcomes.

For descriptive purposes, maternal outcomes were categorized into three levels: 0–3 = good outcome, 4–6 = moderate outcome, and 7–10 = poor outcome. These categories were developed by the researchers solely to facilitate interpretation of the study findings and were not based on previously validated clinical cut-off values.

#### 2.5.5. Instrument V: Neonatal Outcome Assessment Checklist

The Neonatal Outcome Assessment Checklist was developed by the researcher following an extensive review of relevant literature and international clinical guidelines concerning immediate neonatal outcomes among infants born to mothers with asthma [5,22,25,26]. The reviewed sources were used to identify clinically relevant neonatal outcomes and to organize them into domains addressing birth, respiratory status, neonatal intensive care, and overall neonatal condition.

The checklist was completed by the researcher once after delivery (posttest only), using the newborns’ medical records and relevant clinical information documented during the hospital stay and before discharge.

The assessment form comprised eight items distributed across four domains. The first domain, Birth Outcomes, included birth weight, Apgar score at 1 min, and Apgar score at 5 min. Birth weight was classified as normal (≥2500 g), low birth weight (<2500 g), or very low birth weight (<1500 g). Apgar scores at 1 and 5 min were categorized as normal (7–10), moderate depression (4–6), or severe depression (0–3).

The second domain, Respiratory Outcomes, assessed the occurrence of respiratory distress after birth and the type of respiratory support required, including no respiratory support, oxygen therapy, continuous positive airway pressure (CPAP), or mechanical ventilation.

The third domain, Neonatal Intensive Care, included neonatal intensive care unit (NICU) admission and duration of NICU stay, categorized as less than 24 h, 1–3 days, or more than 3 days.

The fourth domain, Overall Neonatal Condition, assessed the neonatal clinical status at discharge and was classified as stable/healthy, complications requiring follow-up, or critical condition.

Scoring system: Each item in the Neonatal Outcome Assessment Checklist was coded for quantitative analysis. A score of 0 indicated a normal or favorable neonatal condition, whereas a score of 1 indicated the presence of an adverse neonatal outcome according to the predefined coding criteria for each variable. The total neonatal outcome score was calculated by summing the scores of the eight items, yielding a possible score ranging from 0 to 8.

For descriptive purposes, the total score was categorized into three levels: 0–2 = good neonatal outcome, 3–5 = moderate adverse neonatal outcome, and 6–8 = poor neonatal outcome. These categories were developed by the researcher solely to facilitate interpretation of the findings and were not based on previously validated clinical cut-off values.

### 2.6. Instrument Development, Validity, Reliability, and Psychometric Properties

Prior to data collection, the study instruments were evaluated to establish their content validity and assess their reliability and psychometric properties. The development and evaluation process was based on a review of relevant literature and international clinical guidelines, followed by formulation of the instrument items, expert review, revision of the instruments based on expert recommendations, and assessment of their psychometric properties in the study sample.

#### 2.6.1. Content Validity

Content validity of the researcher-developed instruments was evaluated by a panel of five experts in Maternal and Newborn Health Nursing and Family and Community Health Nursing. The experts reviewed the instruments with regard to the clarity, relevance, completeness, appropriateness, and adequacy of the items in representing the intended content domains.

Based on the experts’ feedback, minor modifications were made to the wording of some items to improve clarity and appropriateness. For example, in the Asthma Adherence and Self-Monitoring Questionnaire, the item Use of reliever medication (SABA) was rephrased as How often did you use your reliever medication (SABA)? The revised instruments were then finalized for use in the study.

#### 2.6.2. Assessment of Sampling Adequacy

The KMO measure of sampling adequacy and Bartlett’s Test of Sphericity were performed as preliminary assessments of the inter-item correlations. The KMO values ranged from 0.492 to 0.701, while Bartlett’s Test of Sphericity was statistically significant for all instruments (*p* < 0.001), indicating the presence of correlations among the items. These findings were used only as preliminary information regarding the inter-item correlation structure.

#### 2.6.3. Reliability

The reliability of the study instruments was assessed using Cronbach’s alpha coefficient. The Cronbach’s alpha values ranged from 0.887 to 0.904. Specifically, the Asthma Control Test (ACT) demonstrated a Cronbach’s alpha of 0.898, the Asthma Adherence and Self-Monitoring Questionnaire demonstrated a Cronbach’s alpha of 0.904, the Maternal Outcome Assessment Checklist demonstrated a Cronbach’s alpha of 0.901, and the Neonatal Outcome Assessment Form demonstrated a Cronbach’s alpha of 0.887.

The obtained coefficients indicated high internal consistency of the instruments within the study sample. These results provided evidence of satisfactory internal consistency for the present study.

Because the Maternal Outcome Assessment Checklist and Neonatal Outcome Assessment form included clinically heterogeneous outcomes, the Cronbach’s alpha coefficients were interpreted as evidence of internal consistency within the study sample and were not interpreted as evidence that all items represented a single underlying construct.

#### 2.6.4. Overall Psychometric Properties

The psychometric evaluation of the study instruments included expert assessment of content validity, preliminary assessment using the KMO measure and Bartlett’s Test of Sphericity, and assessment of internal consistency using Cronbach’s alpha coefficients.

### 2.7. Administrative Approvals and Ethical Considerations

Approval from the Committee of Research and Ethics, Faculty of Nursing, Menoufia University, was obtained on 16 August 2023 with number (995). The heads of the Maternal and Child Health (MCH) centers received an official letter from the dean of Menoufia University’s Faculty of Nursing asking that the study be conducted. Official authorization to conduct the study was received from the directors of the settings.

To obtain the pregnant women’s agreement and participation, the researchers introduced themselves and explained the purpose and nature of the study. Ethical considerations, including confidentiality, voluntary participation, and informed consent, were addressed throughout the study. Before participation, each participant was provided with full information about the study aims, methodology, potential risks and benefits, and her right to participate voluntarily and withdraw from the study at any time without penalty. The participants were also allowed to ask questions and clarify any concerns regarding the study. Before obtaining written informed consent, the researcher asked each participant whether she was willing to participate in the study. After confirming her willingness to participate, written informed consent was obtained from each participant before enrollment in the study.

### 2.8. Pilot Study

To evaluate the feasibility, suitability, clarity, and comprehensibility of the study instruments, a pilot study was conducted with 14 women, representing 10% of the total sample. The pilot study demonstrated that the instruments were feasible, understandable, and suitable for administration to the target population. Based on the participants’ feedback, minor revisions were made to the wording and phrasing of some questions to improve their clarity and comprehensibility. These revisions were limited to linguistic modifications and did not involve any changes to the content, domains, structure, scoring system, or underlying constructs measured by the instruments. Therefore, no substantive modification was made to the instruments following the pilot study. To avoid potential contamination and ensure that pilot participants did not influence the main study results, all 14 women who participated in the pilot study were excluded from the final study sample.

### 2.9. Field Organization and Intervention Implementation

To ensure standardized implementation of the study procedures, participants were recruited consecutively from the antenatal clinics of two Maternal and Child Health (MCH) Centers (Qebly and Bahary) in Shebin El-Kom, Menoufia Governorate, according to the predefined inclusion criteria until the required sample size of 140 pregnant women was achieved, with 70 participants allocated to the study group and 70 to the control group.

Following the baseline assessment, eligible participants were allocated to the study or control group according to the MCH Center they attended. Women attending Qebly MCH Center were assigned to the study group, whereas women attending Bahary MCH Center were assigned to the control group. This center-level, non-randomized allocation was adopted to minimize contamination between groups, reduce the exchange of intervention-related information among participants, and facilitate standardized delivery of the intervention. However, because allocation was not randomized, the possibility of selection bias and baseline imbalance due to unmeasured factors could not be completely excluded.

#### 2.9.1. Baseline Assessment and Control of Potential Confounding

Before initiation of the intervention, baseline characteristics were assessed in both groups using identical data collection procedures and standardized assessment tools. Baseline assessment included sociodemographic characteristics, obstetric history, and asthma-related clinical characteristics, including asthma duration, previous hospitalization, previous emergency department visits, treatment adherence, asthma severity, and asthma triggers. The same eligibility criteria, assessment procedures, and data collection instruments were applied to both groups to ensure consistency and minimize the potential for differential measurement.

Asthma severity was assessed at baseline, before initiation of the intervention, based on the physician-documented clinical classification available at the time of enrollment and categorized as mild, moderate, or severe. This classification was documented before exposure to the intervention and applied consistently to participants in both groups.

Baseline sociodemographic, obstetric, and asthma-related characteristics were examined to assess observable differences between the study and control groups and to identify potential confounding variables. Although no statistically significant differences were observed in the measured baseline characteristics, the non-randomized center-level allocation means that residual confounding from unmeasured or inadequately measured characteristics could not be completely excluded.

#### 2.9.2. Intervention and Control Conditions

Women in the control group received routine antenatal care provided by the MCH Center according to usual care procedures. Women in the study group received routine antenatal care in addition to a structured nursing intervention supported by the Airlyn mobile health (mHealth) application.

The nursing intervention consisted of standardized educational and supportive components addressing asthma management during pregnancy. The intervention covered asthma and its management during pregnancy, medication adherence, recognition and avoidance of asthma triggers, recognition of warning signs requiring medical attention, appropriate self-management practices, rhythmic breathing exercises, and the importance of regular antenatal and asthma follow-up. The Airlyn mHealth application was used to reinforce rhythmic breathing exercises.

The intervention was delivered according to a predefined protocol using standardized educational materials, including PowerPoint presentations and researcher-developed educational brochures, together with structured verbal explanations and practical demonstrations. All researchers received standardized training from the principal investigator before commencement of data collection. The training covered the intervention content, delivery procedures, educational sequence, use of the Airlyn application, teaching strategies, and practical demonstrations.

#### 2.9.3. Intervention Fidelity

To maintain intervention fidelity, the same protocol and educational sequence were followed throughout the intervention period. Attendance was documented for each educational session. Participants who missed a scheduled session received an individual make-up session covering the same content to ensure completion of all intervention components and minimize variability in intervention exposure.

### 2.10. Intervention Implementation

The intervention was implemented over eight weeks and consisted of three main phases: baseline assessment and orientation, nursing intervention and follow-up, and reinforcement.

#### 2.10.1. Week 1: Baseline Assessment and Orientation Phase

During the first week, baseline data were collected using the Structured Interviewing Questionnaire, the Asthma Control Test (ACT), and the Asthma Adherence and Self-Monitoring Questionnaire, as described in the study instruments. Following completion of the baseline assessment, participants in the study group received an orientation to the intervention program.

The researchers assisted each participant in downloading and installing the Airlyn mobile health application on their smartphone and explained its main functions, particularly the guided rhythmic breathing exercises. Participants were also provided with a researcher-developed educational brochure containing the main educational topics covered throughout the intervention. This phase took around 60–90 min.

#### 2.10.2. Weeks 2–5: Nursing Intervention Phase

The nursing intervention was developed based on current evidence-based literature and the recommendations of the Global Initiative for Asthma [22]. Four structured weekly educational sessions were delivered individually to each pregnant woman. Each session lasted approximately 60–90 min and was designed to facilitate interaction, discussion, practical training, and individualized counseling.

PowerPoint presentations supported by illustrations, photographs, and clinical examples were used to deliver the educational content. The educational brochure was used as a reference throughout the intervention. Demonstration and return-demonstration techniques were applied when practical skills were introduced.

Each session followed a standardized structure consisting of: welcoming and review of key concepts from the previous session (10–15 min); presentation of new educational content using PowerPoint (20–30 min); practical demonstration, return demonstration, and interactive discussion (20–30 min); and summary of key messages and clarification of participants’ questions (10–15 min).

Session One: Understanding Asthma During Pregnancy

The first session focused on increasing participants’ knowledge and awareness of asthma during pregnancy. The session covered the definition and pathophysiology of asthma, physiological respiratory changes during pregnancy, common asthma symptoms, asthma triggers, warning signs of exacerbation, and the potential effects of poorly controlled asthma on maternal and neonatal outcomes. The importance of maintaining optimal asthma control throughout pregnancy was emphasized.

Session Two: Asthma Self-Management, Medication Adherence, and Self-Monitoring

The second session focused on asthma self-management during pregnancy. Participants received education regarding prescribed asthma medications, medication safety during pregnancy, adherence to controller therapy, appropriate use of rescue medications, recognition of worsening symptoms, and the importance of following an individualized asthma plan.

Correct inhaler technique was demonstrated by the researchers using placebo inhalers. Each participant subsequently performed a return demonstration until the correct inhalation technique was achieved.

Session Three: Lifestyle Modification, Trigger Avoidance, and Rhythmic Breathing Exercises

The third session addressed lifestyle modifications that support asthma control during pregnancy. Participants were educated about avoiding common asthma triggers, including tobacco smoke, household dust, air pollution, strong odors, respiratory infections, cold air, allergens, and emotional stress. Additional recommendations included maintaining a healthy diet, adequate hydration, sufficient sleep, appropriate physical activity, stress management, and adherence to scheduled antenatal visits.

During this session, participants received practical training in the use of the Airlyn^®^ mobile application (https://airlyn.io/?utm on 3 June 2025) for guided breathing exercises. The researchers demonstrated how to use the application and supervised each participant while practicing the exercises to ensure correct technique. The application complemented the nurse-led intervention by reinforcing the breathing techniques taught during the educational session and providing participants with a structured means of practicing the exercises independently at home between sessions (2–3 times). Airlyn^®^ was used solely as a supportive educational tool for breathing-exercise training and did not replace nursing education, clinical assessment, or medication management. Participants were instructed to practice the breathing exercises two to three times daily.

Session Four: Reinforcement, Emergency Preparedness, and Asthma Self-Management

The fourth session reinforced the information presented during the previous sessions and focused on recognizing early signs of asthma deterioration, maintaining medication adherence, avoiding triggers, continuing breathing exercises, and attending regular antenatal and asthma follow-up appointments.

Participants were also educated about situations requiring urgent medical attention. Common difficulties encountered during asthma management in pregnancy were discussed, and individualized counseling was provided according to each participant’s needs.

#### 2.10.3. Weeks 6–8: Follow-Up and Reinforcement Phase

During weeks 6–8, participants continued their routine antenatal care and maintained regular use of the Airlyn mobile health application for guided rhythmic breathing exercises.

Weekly follow-up was conducted by the researchers through telephone calls and WhatsApp messages to answer participants’ questions, reinforce previously delivered educational messages, encourage medication adherence, monitor asthma-related symptoms, and provide continued support throughout the intervention period.

#### 2.10.4. Post-Intervention Evaluation Phase

**Figure 1:**
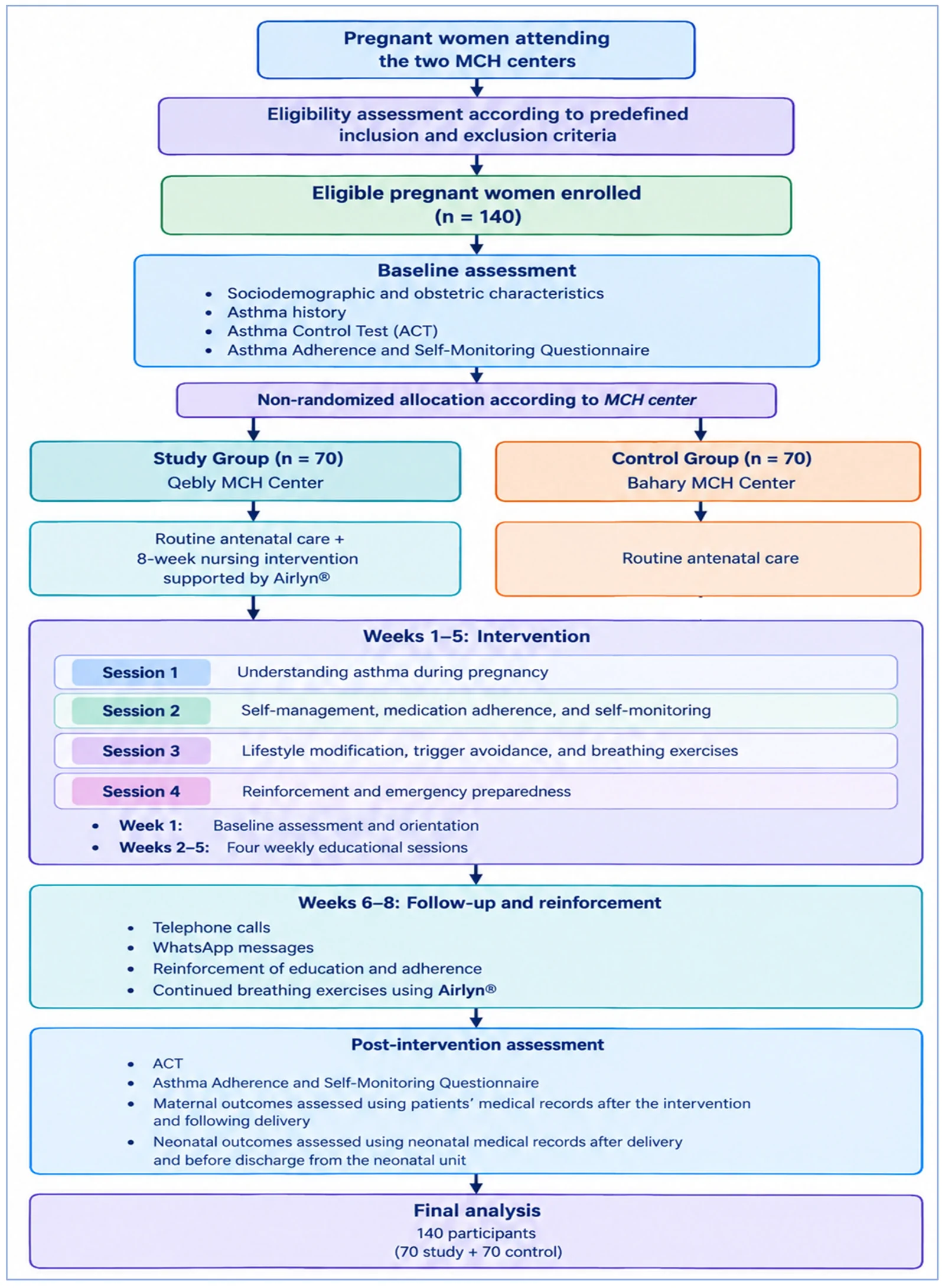
Overall study workflow.

At the end of the eight-week intervention period, asthma control was reassessed using the Asthma Control Test (ACT), and asthma adherence and self-monitoring were reassessed using the Asthma Adherence and Self-Monitoring Questionnaire. Maternal outcomes were assessed using the Maternal Outcome Assessment Checklist, based on information obtained from patients’ medical records following the intervention and following delivery. Following childbirth, neonatal outcomes were assessed using the Neonatal Outcome Assessment Checklist, based on information extracted from newborn medical records after delivery and before discharge from the neonatal unit. The collected data were used to evaluate the effect of the nursing intervention supported by the Airlyn mobile health application in improving asthma control, medication adherence and self-monitoring, and maternal and neonatal outcomes among pregnant women with asthma (Table 2 and Figure 1).

### 2.11. Statistical Analyses

All statistical analyses were performed using SPSS for Windows version 20.0 (SPSS Inc., Chicago, IL, USA). Statistical tests were selected according to the distribution and measurement level of each variable. Continuous variables were summarized as mean ± standard deviation (SD) because they were approximately normally distributed, as confirmed by the Shapiro–Wilk test; therefore, parametric tests were considered appropriate. The independent-samples Student’s *t*-test was used to determine whether statistically significant differences existed between the mean scores of the Case and Control groups for continuous outcomes, including ACT, Adherence and self-monitoring, and Maternal and Neonatal Outcome scores. Cohen’s d was calculated as the difference between group means divided by the pooled standard deviation to quantify the magnitude of between-group differences independently of sample size; values of 0.2, 0.5, and 0.8 were interpreted as small, medium, and large effects, respectively. Ninety-five percent confidence intervals (95% CIs) for the mean differences were reported to indicate the precision of the estimates. Categorical variables, including ACT, Adherence, Maternal Outcome, and Neonatal Outcome category/level classifications, were expressed as frequencies and percentages and compared between groups using the chi-square test. Fisher’s exact test was used when more than 20% of the cells had an expected count below 5. For the ACT, the total score was analyzed as a continuous variable, whereas ACT control levels were analyzed as categorical variables. Pearson’s correlation coefficient (r) was used to assess the direction and strength of linear associations between continuous variables (e.g., total outcome scores). Absolute r values of 0.10–0.29, 0.30–0.49, and ≥0.50 were interpreted as weak, moderate, and strong correlations, respectively. The internal consistency (reliability) of the study questionnaires was assessed using Cronbach’s alpha coefficient, while Sampling Adequacy was evaluated using the Kaiser–Meyer–Olkin (KMO) and Bartlett’s test of sphericity. A two-sided *p*-value < 0.05 was considered statistically significant; significant values are indicated in the tables using * (*p* < 0.05) and ** (*p* < 0.001).

## 3. Results

### 3.1. Sociodemographic, Obstetric, and Asthma History Characteristics

Table 3 illustrates the socio-demographic characteristics of the studied groups. The findings revealed that the highest proportion of women in both groups were aged 25–29 years, had secondary or university education, and resided in urban areas. More than half of the participants in both groups were housewives. No statistically significant differences were observed in any sociodemographic characteristics in both groups (*p* > 0.05).

Table 4 shows the obstetric history of the studied groups. The findings revealed that the study and control groups were similar regarding gravidity, parity, history of abortions, and gestational age, with no statistically significant differences observed (*p* > 0.05). In both groups, more than half of study participants reported a history of abortion; however, the difference was not statistically significant (*p* >0.05).

Table 5 presents the asthma-related characteristics of the studied groups. The findings revealed no statistically significant differences between the study and control groups regarding duration of asthma, history of asthma hospitalization, emergency department visits, treatment adherence, or asthma severity (*p* > 0.05). The distribution of asthma severity was comparable between the study and control groups, with mild asthma reported in 42.9% and 40.0% of participants, moderate asthma in 35.7% and 37.1%, and severe asthma in 21.4% and 22.9%, respectively (*p* = 0.941). In both groups, the highest proportion of participants had asthma for 2–5 years and demonstrated irregular adherence to asthma treatment. Regarding asthma triggers, no statistically significant differences were observed between the two groups (*p* > 0.05).

**Figure 2:**
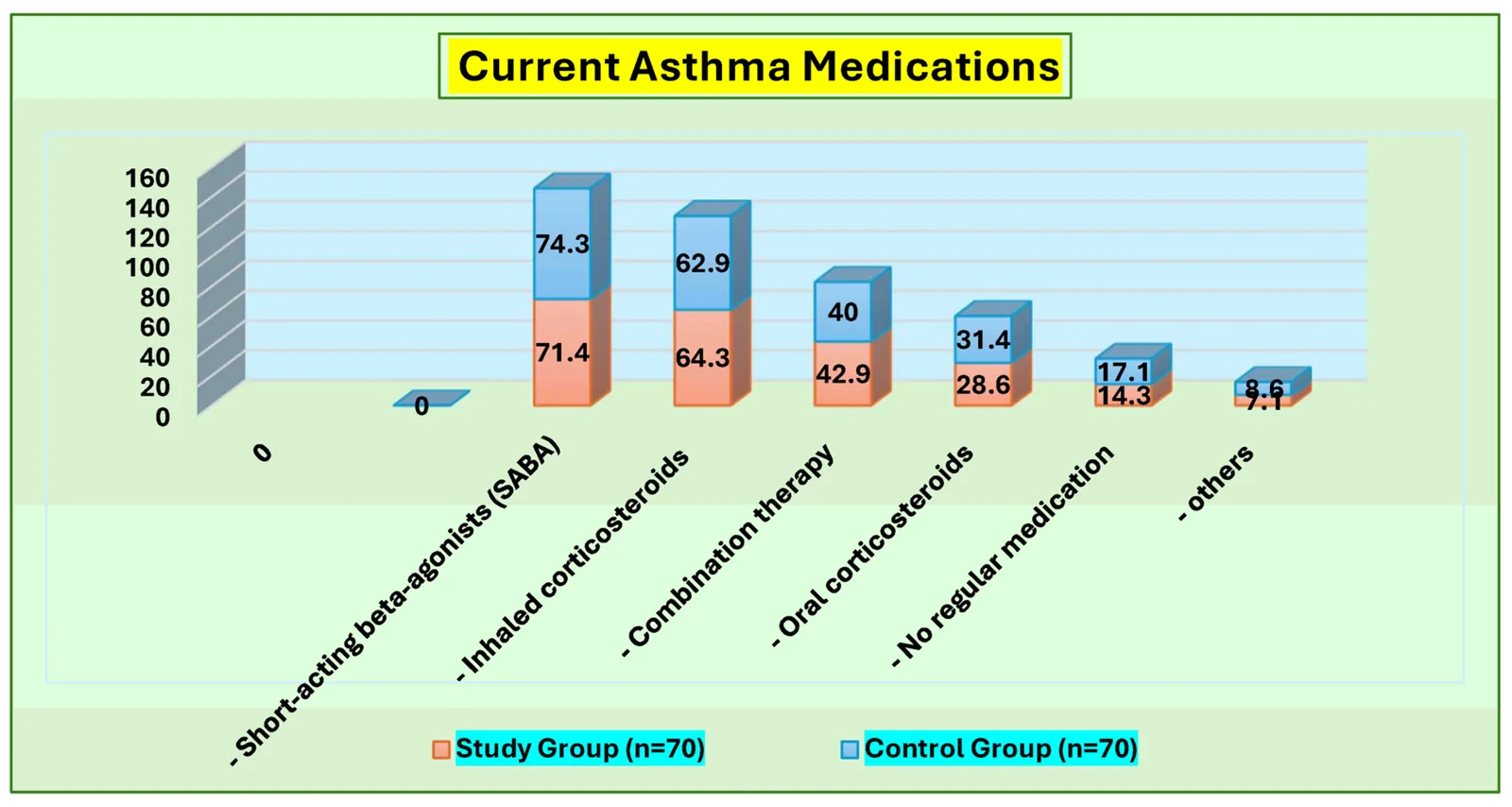
Comparison of Current Asthma Medications among the Studied Group (*N* = 140).

Figure 2 illustrates the current asthma medications used among the studied groups. The findings revealed that short-acting beta-agonists (SABA) and inhaled corticosteroids were the most used medications in both groups. Combination therapy was also reported among a considerable proportion of participants, while smaller percentages used oral corticosteroids or had no regular medication. Only a few participants in both groups reported using other medications.

### 3.2. Asthma Control and Treatment Adherence Outcomes

Table 6 illustrates the comparison of the Asthma Control Test (ACT) overall score between the studied groups before and after the intervention. The findings revealed that most participants in both groups had uncontrolled asthma at the pre-test phase, with no statistically significant difference observed between the groups (*p* > 0.05). Following the intervention, a marked improvement in asthma control was observed in the study group, where nearly two-thirds achieved well-controlled asthma, compared with only a small proportion in the control group. This difference was statistically highly significant (*p* < 0.001). Moreover, the intervention produced a large effect size (Cohen’s d = 1.147), indicating a substantial improvement in asthma control.

Table 7 shows the comparison of asthma adherence and self-monitoring overall score and level between the studied groups before and after the intervention. There was no statistically significant difference between the study and control groups at pre-test (*p* > 0.05), with comparable mean scores (10.1 ± 2.7 vs. 9.3 ± 3.2), indicating similar baseline levels. At post-test, a statistically significant improvement was observed in the study group compared to the control group (*p* < 0.01). The study group showed good adherence (71.4.0%) and poor adherence cases (10.0%), with an improved mean score (5.6 ± 2.7). In contrast, the control group showed minimal adherence (15.7% good adherence, 44.3% poor adherence, mean 9.1 ± 3.1). Additionally, the intervention produced a large effect size (Cohen’s d = 1.199); therefore, the intervention had a positive effect on asthma adherence and self-monitoring in the study group.

### 3.3. Maternal Outcomes

Table 8 presents the comparison of maternal outcomes between the study and control groups after the intervention. The results show that most maternal complications were lower in the study group than in the control group, with statistically significant differences in gestational hypertensive disorders (37.1% vs. 57.1%), gestational diabetes mellitus (31.4% vs. 50.0%), urinary tract infection (34.3% vs. 64.3%), preterm labor/PPROM (37.1% vs. 61.4%), antepartum hemorrhage (38.6% vs. 61.4%), postpartum hemorrhage (35.7% vs. 62.9%), and venous thromboembolism (41.4% vs. 64.3%). A statistically significant difference was also observed in the mode of delivery (*p* < 0.001), with vaginal delivery being more common in the study group (61.4%) than in the control group (24.3%). However, no statistically significant difference was found between the two groups regarding respiratory infections during pregnancy (42.9% vs. 58.6%, *p* = 0.063).

Table 9 shows the comparison of total maternal outcomes between the study and control groups after the intervention. There was a highly statistically significant difference between the two groups (*p* < 0.001). The study group demonstrated better maternal outcomes, with 72.9% of participants having a good outcome, 17.1% having a moderate outcome, and 10.0% having a low outcome, with an improved mean score (3.5 ± 1.7). In contrast, the control group showed poorer outcomes, as 17.1% had a good outcome, 40.0% had a moderate outcome, and 42.9% had a low outcome, with a mean score of 6.1 ± 2.6. Moreover, the intervention produced a large effect size (Cohen’s d = 1.174), indicating that the intervention had a positive effect on total maternal outcomes in the study group compared to the control group.

### 3.4. Neonatal Outcomes

Table 10 presents the comparison of neonatal outcomes between the study and control groups after the intervention. The findings demonstrated statistically significant differences between the study and control groups regarding birth weight, Apgar scores at 1 and 5 min, respiratory distress after birth, and NICU admission (*p* < 0.05). The study group showed a higher proportion of neonates with normal birth weight (71.4% vs. 52.9%), normal Apgar scores at 1 min (64.3% vs. 44.3%) and 5 min (70.0% vs. 35.7%), as well as a higher proportion of neonates without respiratory distress after birth (75.7% vs. 51.4%) and without NICU admission (75.7% vs. 48.6%) compared with the control group. However, no statistically significant differences were observed between the two groups regarding the type of respiratory support required (*p* = 0.062), duration of NICU stay (*p* = 0.081), or neonatal outcome status at discharge (*p* = 0.075).

Table 11 shows the comparison of total neonatal outcomes between the study and control groups after the intervention. A highly statistically significant difference was observed between the two groups (*p* < 0.001). The study group demonstrated better neonatal outcomes, where 71.4% of neonates had a good outcome, 24.3% had a moderate outcome, and 4.3% had a low outcome, with an improved mean score (7.7 ± 2.2). In contrast, the control group showed poorer outcomes, as only 24.3% had a good outcome, while the majority (61.4%) had a moderate outcome and 14.3% had a low outcome, with a mean score of (5.5 ± 2.1). Moreover, the intervention produced a large effect size (Cohen’s d = 1.022), indicating the intervention had a positive effect in improving total neonatal outcomes in the study group compared to the control group.

### 3.5. Correlation Analyses

**Figure 3:**
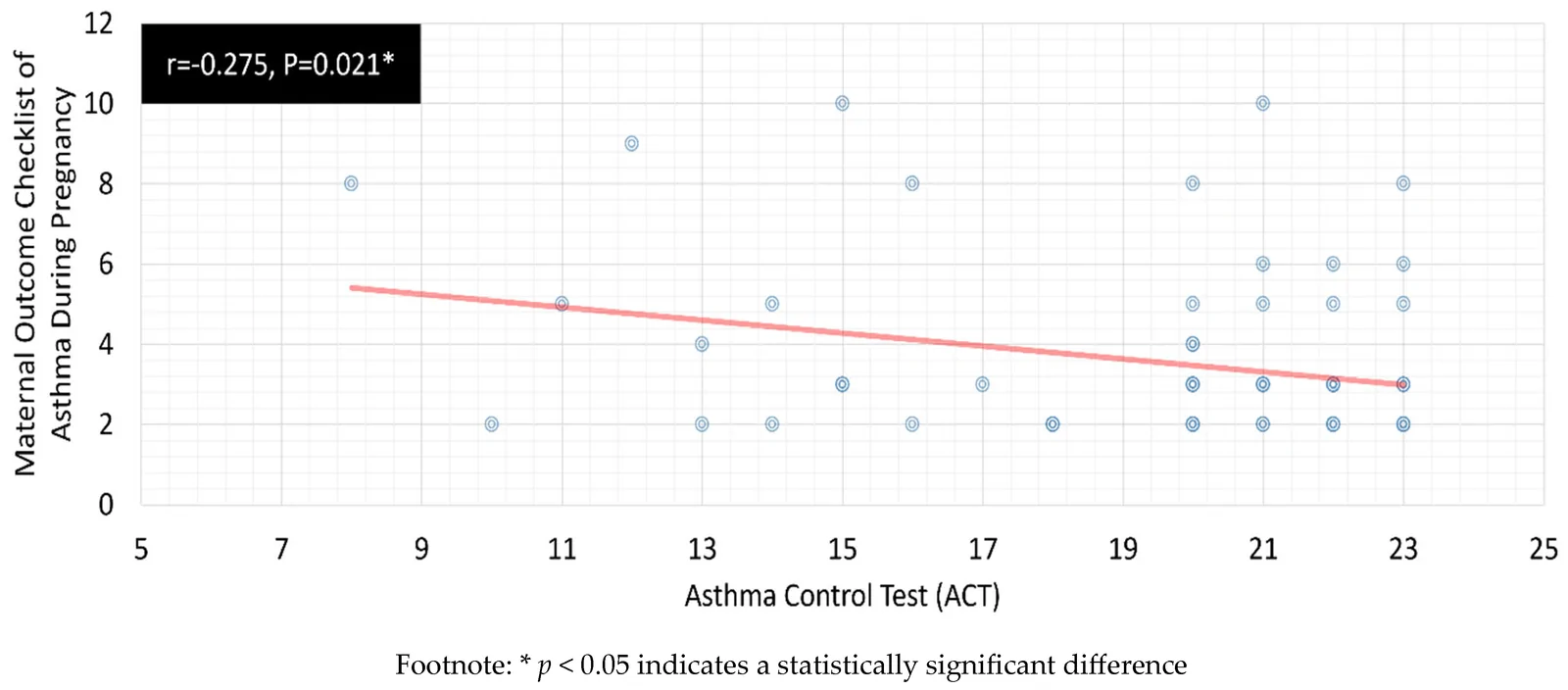
Correlation between Total Maternal Outcomes and Asthma Control Test Score.

Figure 3 illustrates the correlation between adverse maternal outcomes and total asthma control test scores. The results show a slight negative correlation between adverse maternal outcomes and the total asthma control test. The Coefficient of Determination (R^2^) value of 0.275 indicates that as the asthma control test score increases, the maternal outcome score decreases.

**Figure 4:**
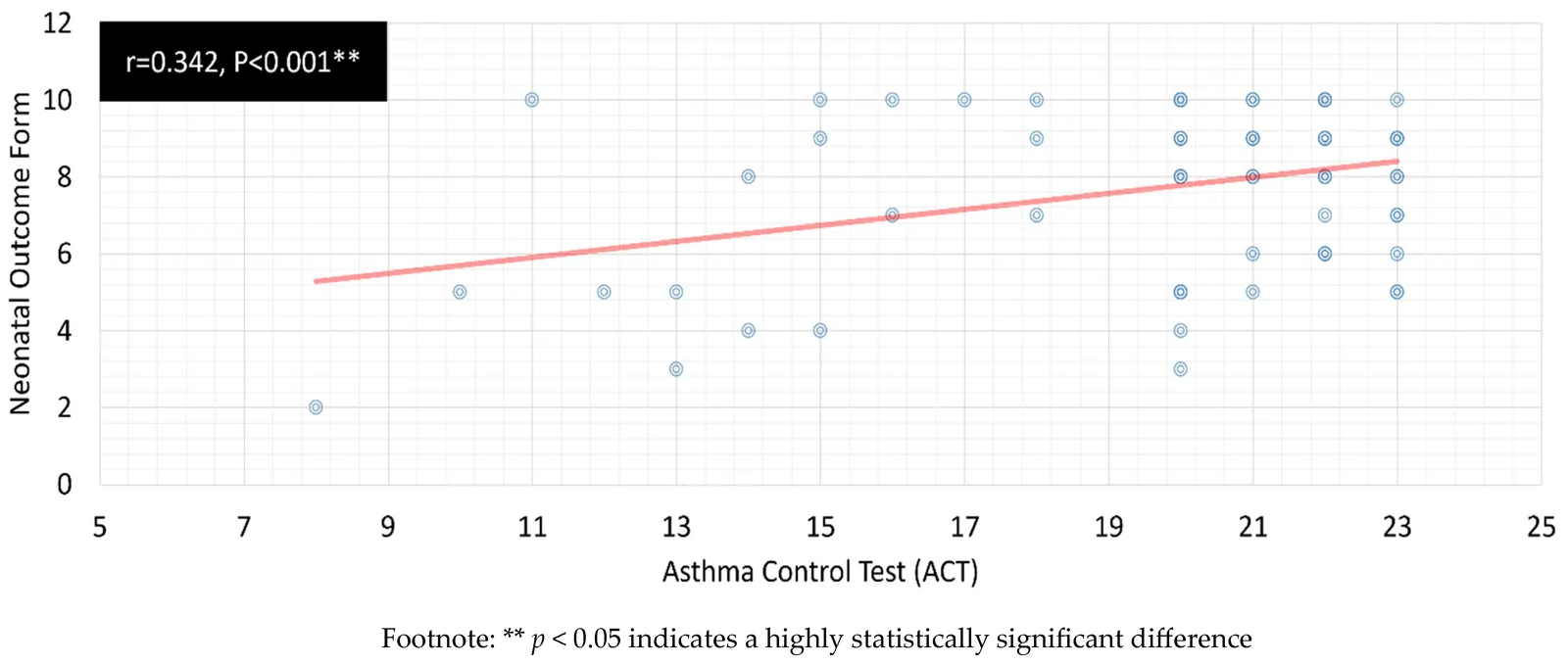
Correlation between Total Newborn Outcomes and Total Asthma Control Test Scores.

Figure 4 illustrates the correlation between total Asthma Control Test (ACT) scores and total newborn outcome scores. The results show a positive correlation (r = 0.342, *p* < 0.001), indicating that better asthma control was associated with better neonatal outcomes.

**Figure 5:**
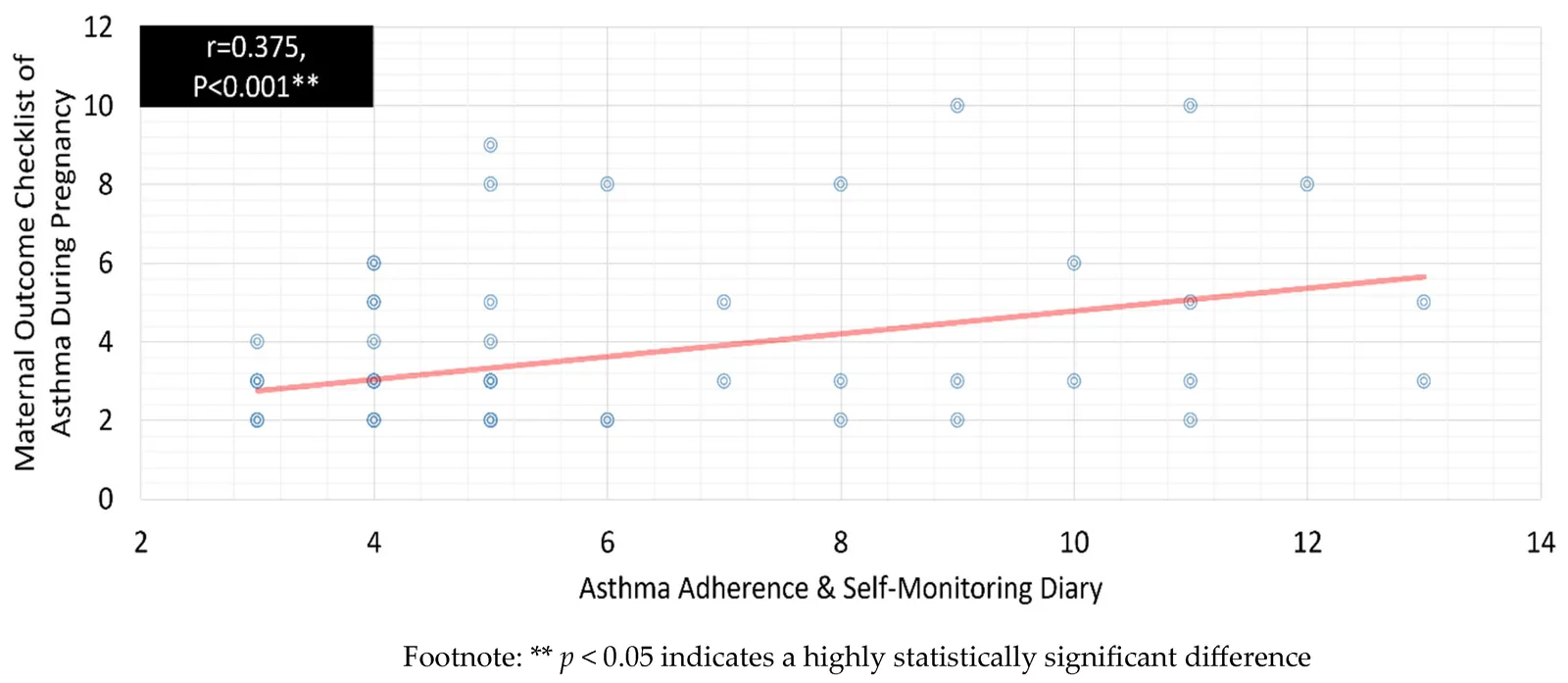
Correlation between Total Maternal Outcomes and Total Asthma Adherence and Self-Monitoring Score.

Figure 5 illustrates the correlation between total maternal outcomes and asthma adherence and self-monitoring scores. The results show a positive correlation. The Coefficient of Determination (R^2^) value of 0.375, *p* < 0.001, indicates that higher asthma adherence and self-monitoring scores were associated with better maternal outcomes.

**Figure 6:**
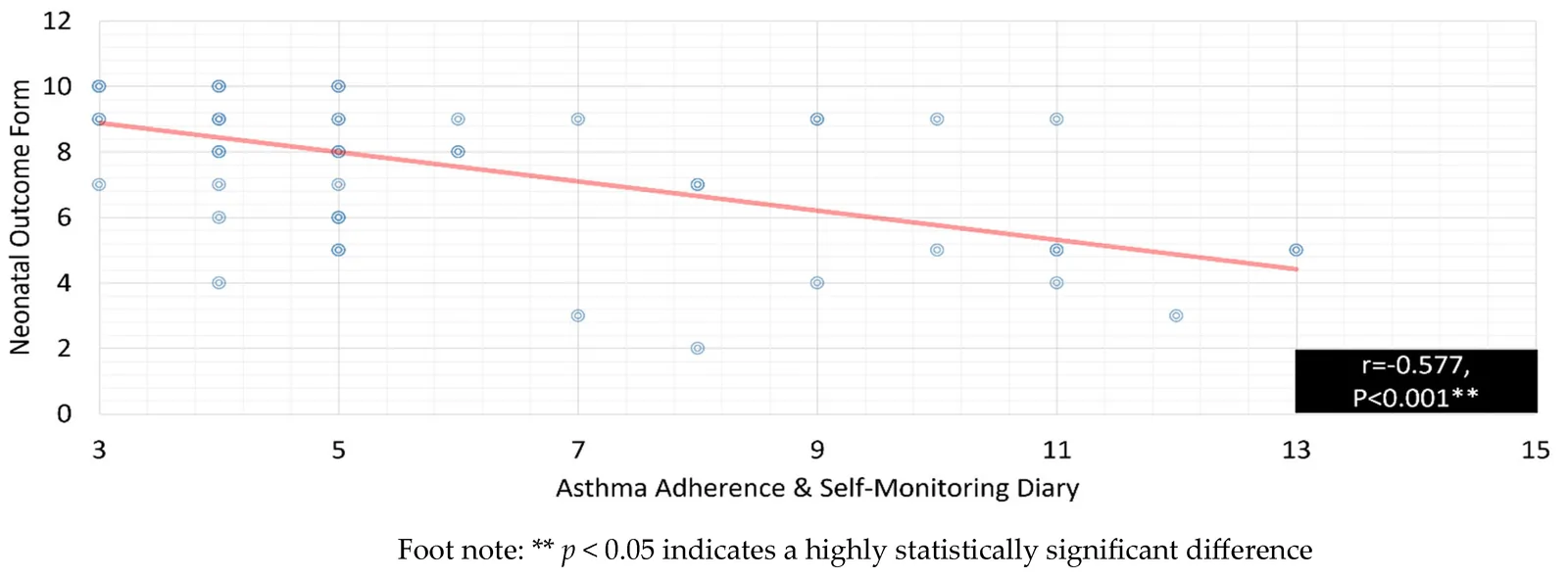
Correlation of Total Newborn Outcomes with Asthma Adherence and Self-Monitoring Scores.

Figure 6 illustrates the correlation between total neonatal outcomes and asthma adherence and self-monitoring scores. The results show a moderate negative correlation. The Coefficient of Determination (R^2^) value = −0.577, *p* < 0.001, indicating that higher asthma adherence and self-monitoring scores were associated with fewer adverse neonatal outcomes.

## 4. Discussion

### 4.1. Asthma History and Clinical Characteristics

Asthma is the most common chronic medical disorder affecting pregnancy. Poor asthma control has been associated with increased risks for both the mother and fetus, including perinatal mortality, intrauterine growth restriction, preeclampsia, preterm birth, and low birth weight. Therefore, maintaining adequate asthma control throughout pregnancy is considered essential to promote maternal and fetal health [26]. Accordingly, the present study was designed to investigate the effect of a nursing intervention supported by mobile health (mHealth) applications on asthma control and maternal and neonatal outcomes among pregnant women with asthma.

Regarding the asthma history of the participants, the findings showed that the study and control groups were comparable in terms of asthma duration, previous asthma-related hospitalization, emergency department visits due to asthma, adherence to asthma treatment, and asthma severity, with no statistically significant differences between the groups. These findings are consistent with Ibrahim et al. (2019) [27], who likewise reported no statistically significant differences among participants regarding asthma history, clinical symptoms, disease duration, treatment, hospital admissions, and physician visits.

Moreover, most participants in both groups had experienced asthma for 2–5 years and demonstrated irregular adherence to their prescribed asthma medications. This finding is consistent with Rey, Chełmińska, and Damps-Konstańska (2024) [28], who also reported that many pregnant women with asthma were exposed to different triggering factors and discontinued their regular asthma treatment after confirming pregnancy. Although irregular adherence to asthma treatment was common among participants in the present study, the underlying reasons for non-adherence were not investigated. Concerns regarding the safety of asthma medications during pregnancy may represent one possible explanation.

### 4.2. Asthma Medications and Triggers

With regard to current asthma medications, the present study showed that short-acting beta-agonists (SABA) and inhaled corticosteroids were the medications most frequently used in both the study and control groups. By comparison, only a small proportion of participants reported using oral corticosteroids. These findings are comparable to those reported by Rohn et al. (2023) [29] and Palmsten et al. (2016) [30], who similarly found that short-acting β2-agonists and inhaled corticosteroids were the most commonly prescribed asthma medications during pregnancy.

Regarding asthma triggers, the present study found that a large proportion of participants in both groups reported exposure to asthma-triggering factors. Strong odors and chemical irritants were reported more frequently by participants in the study group, whereas respiratory infections were more common among those in the control group. Comparable proportions of participants in both groups identified allergens, weather changes or cold air, physical exertion, and emotional stress as asthma triggers. These findings are consistent with Al Jerdabi et al. (2025) [31], who reported that these factors were frequently associated with poorer asthma control. These findings highlight the importance of educating pregnant women with asthma about common triggers and appropriate strategies to avoid or minimize exposure to them as part of asthma self-management.

### 4.3. Effect of the mHealth-Supported Nursing Intervention on Asthma Control

The findings of the present study showed that the nursing intervention supported by mobile health (mHealth) applications was associated with improved asthma control among pregnant women with asthma. Before the intervention, the majority of women in both the study and control groups had uncontrolled asthma, with no statistically significant differences between the groups, indicating comparable baseline status. Following the intervention, nearly two-thirds of participants in the study group achieved well-controlled asthma, whereas only a small proportion of those in the control group reached adequate asthma control. These findings suggest that the mHealth-supported nursing intervention may have contributed to improved asthma control. However, given the quasi-experimental design, the observed association should be interpreted with caution, and a causal relationship cannot be definitively established.

The observed improvement in asthma control may reflect several components of the nursing intervention, including individualized education and mobile health (mHealth) support. The intervention encouraged adherence to prescribed medications, reinforced appropriate inhaler technique, promoted regular symptom monitoring, increased awareness of asthma triggers, and facilitated self-management through continuous education and reminders.

With regard to the comparison of total Asthma Control Test (ACT) scores between the study and control groups before and after the intervention, the findings showed that the majority of participants in both groups had uncontrolled asthma at baseline, with no statistically significant differences between the groups. After the intervention, asthma control improved significantly in the study group, with nearly two-thirds of participants achieving well-controlled asthma, whereas only a small proportion of women in the control group reached this level of control. These findings were consistent with those of Gebresilassie, Worku, Ahmed, and Kabeta (2025) [20], who reported that most participants in both the intervention and comparison groups had poor asthma control before the intervention. Following the intervention, asthma control scores were significantly higher in the intervention group than in the comparison group. Also, these results were congruent with Mohamed, Mohammed and Khalaf (2025) [10], who reported that there was a statistically significantly higher mean of asthma-control tests, adherence score, and quality-of-life questionnaire among the intervention group compared to the control group after 3 months of intervention. Additionally, there were significantly fewer exacerbations and hospitalizations in the intervention group.

The present findings are also comparable to those reported by Murphy et al. (2005) [8], who evaluated a nurse-led asthma self-management education program during antenatal care. The intervention included education on medication adherence, asthma knowledge, proper inhaler technique, self-monitoring, and written asthma action plans. The authors suggested that these intervention components were associated with improved asthma management during pregnancy and might contribute to better maternal and neonatal outcomes. However, they acknowledged that the absence of a comparison group limited causal inference. Similarly, the present findings support a possible association between structured nursing education and improved asthma control, while recognizing that causality cannot be confirmed.

Likewise, Murphy (2015) [32] reported that individualized asthma education delivered by specialist asthma nurse educators was associated with improvements in self-management behaviors among pregnant women with asthma. After the educational intervention, self-reported non-adherence to inhaled corticosteroids decreased from 40% to 21%, while incorrect inhaler technique declined from 16% to 4%. Additionally, women with severe asthma experienced reductions in nighttime symptoms and the use of reliever medications. These findings are broadly consistent with the present study and support the potential value of nurse-led educational interventions for asthma management during pregnancy. Likewise, Ameyaw, Amoah, and Ezezika (2024) [33] concluded that most mHealth apps were implemented by sending SMS text messages with mobile phones. mHealth interventions were most effective in 5 areas: maternal anxiety and depression, diabetes in pregnancy, gestational weight management, maternal health care use, behavioral modification toward smoking cessation, and controlling substance use during pregnancy.

The findings of the present study are further supported by Grzeskowiak et al. (2016) [34], who found that pregnant women receiving structured asthma self-management education achieved significantly greater improvements in Asthma Control Questionnaire (ACQ) scores than those receiving routine antenatal care. The authors also reported that recurrent uncontrolled asthma was more strongly associated with adverse pregnancy outcomes than isolated asthma exacerbations, emphasizing the importance of maintaining asthma control throughout pregnancy. Likewise, Bonham, Patterson, and Strek (2018) [2] found that women who participated in the Multidisciplinary Approach to Management of Maternal Asthma (MAMMA) intervention demonstrated greater improvement in Asthma Control Test (ACT) scores than those receiving standard care. Taken together, these findings are consistent with the results of the present study and suggest that structured nursing interventions supported by mHealth technology may be associated with improved asthma control during pregnancy. Nevertheless, confirmation through well-designed randomized controlled trials is warranted.

In addition, the present findings are comparable to those reported by Thomas et al. (2025) [19], who observed a significant improvement in the overall Asthma Control Questionnaire (ACQ) score, with a larger proportion of participants achieving well-controlled asthma at follow-up than at baseline. The authors attributed these findings to the implementation of asthma self-management strategies, appropriate medication use, and avoidance of asthma triggers.

### 4.4. Effect of the mHealth-Supported Nursing Intervention on Treatment Adherence and Self-Monitoring

Regarding the comparison of total mean scores for asthma treatment adherence and self-monitoring between the study and control groups before and after the intervention, the findings showed no statistically significant differences between the two groups at baseline, reflecting comparable levels of treatment adherence and self-monitoring. Following the intervention, however, the study group demonstrated significantly higher adherence and self-monitoring scores than the control group. These findings are consistent with those of Sebhi et al. (2025) [35], who likewise reported significant improvements in treatment adherence and self-monitoring following an educational intervention. Although improved adherence and self-monitoring may have contributed to better asthma management, the present study was not designed to determine whether these factors directly accounted for the observed improvements in asthma control.

### 4.5. Maternal Outcomes

With respect to maternal outcomes following the intervention, maternal complications were generally less frequent in the study group than in the control group, with a statistically significant difference observed for the majority of maternal outcomes, including antepartum hemorrhage, Postpartum hemorrhage, Preterm labor, and the mode of delivery, with vaginal delivery occurring more frequently among women in the study group. These findings are consistent with Ibrahim et al. (2019) [27], who reported lower rates of several maternal complications and higher rates of vaginal delivery among women who participated in an asthma education program. Although improved asthma self-management may have contributed to these findings, the present study does not permit conclusions regarding a direct causal relationship between the intervention and maternal outcomes because of its quasi-experimental design.

Regarding the comparison of overall maternal outcomes between the study and control groups following the intervention, women in the study group demonstrated more favorable maternal outcomes than those in the control group, and the difference was highly statistically significant. These findings are comparable to those reported by Haastrup et al. (2026) [36], who found that after the intervention, the majority of the intervention group had good perinatal outcomes compared to pregnant women with asthma allocated to usual care, with a statistically significant difference. Also, Er et al. (2026) [37] supported that the mHealth interventions used included mobile apps, web-based platforms, smart bands, and short message service, with interventions showing positive effects on maternal outcomes. Enhanced self-management skills, improved inhaler technique, and better treatment adherence may have been associated with these outcomes.

### 4.6. Neonatal Outcomes

With respect to neonatal outcomes, neonates born to mothers in the intervention group demonstrated significantly better outcomes than those born to mothers in the control group. Significant differences were observed in birth weight, Apgar score, respiratory distress after birth, and neonatal intensive care unit (NICU) admission; however, no statistically significant differences were found in type of respiratory support required, the duration of NICU admission, and neonatal outcome status at discharge.

The more favorable neonatal outcomes observed in the intervention group may be associated with the better maternal asthma control observed following the intervention. Previous studies have suggested that adequate asthma control during pregnancy is associated with improved placental oxygenation, fewer asthma exacerbations, and a lower likelihood of adverse pregnancy outcomes. However, the present study did not directly assess these biological mechanisms, and therefore this interpretation should be considered cautiously.

These findings are consistent with the meta-analysis by Murphy et al. (2011) [4], which reported that active asthma management during pregnancy was associated with a reduced risk of adverse perinatal outcomes. Similarly, Georgakopoulou et al. (2024) [38] reported lower risks of adverse neonatal outcomes following treatment initiation. Furthermore, Brightling (2020) [39] and Grzeskowiak et al. (2016) [34] emphasized the association between optimal asthma control during pregnancy and improved neonatal outcomes. Collectively, these studies are consistent with the present findings and suggest that interventions promoting asthma control may be associated with more favorable maternal and neonatal outcomes. Nevertheless, because the present study employed a quasi-experimental design, these findings should be interpreted with caution, and further randomized controlled trials are required to confirm these associations and clarify the underlying mechanisms.

Regarding the comparison of overall neonatal outcomes between the study and control groups following the intervention, the findings of the present study showed that the study group achieved more favorable neonatal outcomes, with most neonates demonstrating good outcomes and nearly one-quarter showing moderate outcomes with an improved mean score (7.7 ± 2.2), whereas the control group had poorer neonatal outcomes with a mean score of (5.5 ± 2.1). A highly statistically significant difference was identified between the two groups (*p* < 0.001). These findings are consistent with those of Yland et al. (2020) [40], who found that women with well-controlled asthma had lower relative risks of stillbirth, spontaneous abortion, preterm birth, small-for-gestational-age (SGA), neonatal intensive care unit (NICU) admission, and congenital malformations than women with poorly controlled asthma. Women with poor control late in pregnancy had an increased risk of preterm birth (relative risk, 1.39; 95% CI, 1.32–1.46) and NICU admission (relative risk, 1.26; 95% CI, 1.17–1.35). More severe asthma was associated with SGA (relative risk, 1.18; 95% CI, 1.07–1.30). Although greater adherence to the nurse-led antenatal asthma management program may have contributed to the more favorable neonatal outcomes observed in the present study, the quasi-experimental design does not allow a definitive conclusion that the intervention directly caused these outcomes. Other unmeasured factors may also have influenced the results.

### 4.7. Associations Between Asthma Control and Maternal and Neonatal Outcomes

With respect to the correlation between overall maternal outcomes and total Asthma Control Test (ACT) scores, the present study identified a slight negative correlation, indicating that higher ACT scores were associated with fewer adverse maternal outcomes. These findings are in agreement with Haastrup et al. (2026) [36], who also reported an inverse association between poor asthma control during pregnancy and adverse maternal outcomes. However, the observed correlation should not be interpreted as evidence of a causal relationship, as correlation does not establish causation.

Regarding the correlation between overall neonatal outcomes and total Asthma Control Test (ACT) scores, the findings demonstrated a slight negative correlation, indicating that higher ACT scores were associated with fewer adverse neonatal outcomes. These findings are consistent with those of Robijn et al. (2024) [6], who likewise reported an inverse relationship between maternal asthma control and adverse neonatal outcomes. Although these findings suggest an association between better asthma control and more favorable neonatal outcomes, the present study cannot determine whether improved asthma control directly resulted in these outcomes.

### 4.8. Associations Between Treatment Adherence, Self-Monitoring, and Maternal and Neonatal Outcomes

With regard to the correlation between overall maternal outcomes and asthma treatment adherence and self-monitoring scores, the present study demonstrated a positive correlation, indicating that higher adherence and self-monitoring scores were associated with better maternal outcomes. These findings are consistent with Georgakopoulou et al. (2024) [38], who reported positive associations between asthma management behaviors and favorable pregnancy outcomes. Nevertheless, the correlational nature of these findings precludes conclusions regarding the direction or causality of these relationships.

Regarding the correlation between overall neonatal outcomes and asthma treatment adherence and self-monitoring scores, the present study demonstrated a positive correlation, indicating that better adherence and higher self-monitoring scores were associated with more favorable neonatal outcomes. These findings are consistent with those of Colas et al. (2025) [41], who reported similar associations between adherence to asthma management and neonatal outcomes. While these findings support a potential relationship between adherence, self-monitoring, and neonatal health, they should be interpreted cautiously because other maternal or clinical factors not examined in the present study may also have contributed to the observed outcomes (Table S1).

### 4.9. Overall Interpretation of the Findings

Overall, the findings of the present study suggest that a structured nursing intervention supported by mobile health (mHealth) applications was associated with improved asthma control and more favorable maternal and neonatal outcomes among pregnant women with asthma. The intervention, which incorporated individualized education, symptom monitoring, medication adherence support, and ongoing professional follow-up, may have facilitated better self-management behaviors. However, because this study employed a quasi-experimental design, the observed associations should be interpreted with caution, and causal inferences cannot be definitively established. Further well-designed randomized controlled studies are warranted to confirm these findings and to clarify the mechanisms through which mHealth-supported nursing interventions may influence maternal and neonatal outcomes.

### 4.10. Study Limitations

This study has several limitations that should be considered when interpreting its findings. First, the quasi-experimental design, convenience sampling technique, and non-randomized allocation of participants according to the Maternal and Child Health (MCH) Center attended may have introduced selection bias and increased the risk of systematic differences between the study and control groups. To assess baseline comparability, sociodemographic, obstetric, and asthma-related characteristics were evaluated using standardized data collection procedures and identical assessment tools before implementation of the intervention. No statistically significant differences were identified in the measured baseline characteristics, supporting the comparability of the groups at baseline. Nevertheless, because allocation was non-randomized, residual confounding due to unmeasured or inadequately measured characteristics cannot be completely excluded. Therefore, the findings should be interpreted with caution, particularly regarding causal relationships. Second, the use of convenience sampling and recruitment from only two specific MCH Centers may limit the external validity and generalizability of the findings to pregnant women with asthma in other populations, healthcare settings, or geographic areas. In addition, the sample size was primarily determined based on the main study comparison and was not specifically powered to detect differences in individual maternal and neonatal outcomes. Third, the maternal and neonatal outcome scores were constructed by combining several heterogeneous clinical outcomes into composite measures. Although these composite scores provided an overall summary of maternal and neonatal outcomes, combining outcomes with different clinical characteristics and levels of clinical importance may limit the interpretation of the overall scores. A change in the composite score may not necessarily reflect a consistent change across all individual outcome components. Therefore, the composite measures should be interpreted cautiously alongside the individual maternal and neonatal outcomes. Fourth, asthma control was assessed using the self-reported Asthma Control Test (ACT), which may be subject to reporting and response biases. Although the ACT is a standardized instrument, self-reported measures may be influenced by participants’ understanding, recall, and perceptions of their symptoms and asthma control. Fifth, maternal and neonatal outcomes were assessed using information extracted from patients’ and newborns’ medical records. Consequently, the accuracy of the findings depended on the completeness and quality of the available medical documentation, and some relevant clinical information may not have been fully captured. Furthermore, neonatal outcomes were assessed only during the immediate postnatal period before hospital discharge; therefore, longer-term neonatal outcomes were not evaluated. Sixth, the adherence and outcome assessment instruments developed by the researcher may require further psychometric validation in larger and independent populations before their wider application. Finally, because the nursing education and mobile health (mHealth) application were delivered as a combined intervention, it was not possible to determine the independent contribution of each component to the observed outcomes.

## 5. Conclusions and Future Work

The present study provides preliminary evidence that an eight-week structured nursing intervention supported by the Airlyn^®^ mobile health (mHealth) application was associated with significant improvements in asthma management among pregnant women with asthma. Compared with routine antenatal care, women who received the intervention demonstrated better asthma control, treatment adherence, and self-monitoring, as well as more favorable maternal and neonatal outcomes. Mean ACT scores increased from 14.5 ± 4.2 at baseline to 19.7 ± 3.6 after the intervention in the study group, compared with 15.2 ± 4.2 in the control group (*p* < 0.001). Moreover, 75.7% of women in the intervention group achieved well-controlled asthma compared with 17.1% in the control group. Treatment adherence and self-monitoring also improved significantly (5.6 ± 2.7 vs. 9.1 ± 3.1, *p* < 0.001). Maternal and neonatal outcomes were significantly more favorable in the intervention group, with mean maternal outcome scores of 3.5 ± 1.7 versus 6.1 ± 2.6 and neonatal outcome scores of 7.7 ± 2.2 versus 5.5 ± 2.1, respectively (both *p* < 0.001).

The main novelty of the study lies in integrating an individualized nursing intervention with mHealth-supported self-management within pregnancy-specific asthma management. The intervention combined asthma education, medication-adherence reinforcement, self-monitoring, inhaler-technique training, trigger avoidance, rhythmic breathing exercises, and continued telephone/WhatsApp follow-up, with Airlyn^®^ serving as a supportive tool for guided breathing practice. Despite these encouraging findings, the results should be interpreted cautiously because of the quasi-experimental design and non-randomized group allocation; therefore, the observed associations should not be interpreted as definitive evidence of causality. Future research should include adequately powered multicenter randomized controlled trials with standardized intervention protocols, concealed allocation where feasible, and longer follow-up to determine whether the observed benefits are sustained throughout pregnancy and after delivery. Future studies should also examine the individual contribution of the intervention components, incorporate objective measures of medication adherence and asthma control where feasible, and assess the acceptability, usability, cost-effectiveness, and long-term clinical value of mHealth-supported asthma care.

Overall, the findings support the potential feasibility and clinical relevance of integrating structured nursing care with mHealth-supported self-management for pregnant women with asthma. However, further rigorous evidence is required before widespread implementation and definitive causal conclusions regarding maternal and neonatal benefits can be established.

## Figures and Tables

**Table 1:** Summary of Previous Relevant Studies.

Reference	Study Population	Intervention Type	Method	Outcome Measures	Main Achieved Results	Limitations
Yoo et al., 2021 [14]	Pregnant women with asthma	Integrated asthma education, monitoring, and specialist management	Prospective intervention study	Asthma control, asthma-related QoL	Improvement in asthma control and QoL	No control group; loss to follow-up
Dhippayom et al., 2022 [15]	Adults with asthma	Self-management and behavioral support, including eHealth	Systematic review and network meta-analysis	Asthma control, healthcare utilization	Behavioral/eHealth support showed potential benefits for asthma management.	Heterogeneity; not pregnancy-specific
Mosnaim et al., 2021 [16]	Patients with asthma	Digital health interventions	Scoping review	Adherence, asthma impairment, engagement	Interactive digital interventions showed potential benefits	Heterogeneous interventions; not pregnancy-specific
Doshi et al., 2021 [17]	Patients with asthma	Technology-based asthma interventions	Systematic review	Patient-reported outcomes	Most studies improved at least one patient-reported outcome	Heterogeneous intervention and outcome measures
Nguyen et al., 2021 [13]	Patients with asthma	mHealth with inhaler sensors	Systematic review	Medication adherence, rescue-inhaler use, asthma control	Improved adherence and reduced rescue-inhaler use; inconsistent effect on asthma control	Limited clinical outcomes; not pregnancy-specific
Schulte et al., 2021 [12]	Patients with asthma/COPD	eHealth education, monitoring, counseling, and self-support	Systematic review	Medication adherence	Mixed effects on adherence	Heterogeneity in interventions and outcome assessment
Chan et al., 2022 [11]	Adults and children with asthma	Digital adherence and self-management interventions	Cochrane systematic review; 40 RCTs, 15,207 participants	Adherence, asthma control, QoL	Improved adherence and probable improvement in asthma control and QoL	Variable certainty; risk of bias and heterogeneity
Farzandipour et al., 2024 [18]	Patients with asthma	Smartphone-based self-management application	Randomized controlled trial	Asthma control, QoL	Significant improvement in asthma control and QoL	Small sample; non-pregnant population
Thomas et al., 2025 [19]	Pregnant women with asthma	Virtual antenatal care with digital resources and smartphone application	Randomized controlled tele-trial	Engagement, asthma control	Greater engagement with virtual care; asthma control improved in both groups	No significant between-group difference in asthma control; limited maternal/neonatal outcomes
Current study	140 pregnant women with physician-diagnosed asthma	Structured nursing intervention supported by mHealth application	Quasi-experimental pretest–post-test with control group	ACT, adherence, self-monitoring, maternal and neonatal outcomes	Significant improvements in asthma control, adherence, self-monitoring, and maternal/neonatal outcomes (*p* < 0.001)	Convenience sample; two centers; quasi-experimental design

**Table 2:** Structured schedule of the eight-week nursing intervention supported by the Airlyn^®^ mHealth application.

Time Point	Session/Phase	Objectives	Nursing Activities	mHealth Component (Airlyn^®^)	Frequency/Duration	Assessment/Follow-Up
Week 1	Baseline assessment and orientation	To establish baseline characteristics and asthma-related status and orient participants to the intervention	Collect sociodemographic and obstetric data. Assess asthma history and clinical characteristics. Administer the ACT and Asthma Adherence and Self-Monitoring Questionnaire. Explain the intervention objectives, procedures, and participant responsibilities. Provide the educational brochure.	-Assist participants in downloading and installing Airlyn^®^. -Explain the main functions of the application.	One individual session; approximately 60–90 min	Baseline assessment using the Structured Interviewing Questionnaire, ACT, and Asthma Adherence and Self-Monitoring Questionnaire.
Week 2	Session 1: Understanding asthma during pregnancy	To improve knowledge and awareness of asthma and the importance of maintaining asthma control during pregnancy	Explain the definition and basic pathophysiology of asthma. Discuss physiological respiratory changes during pregnancy. Identify common asthma symptoms and triggers. Explain warning signs of asthma exacerbation. Discuss potential maternal and neonatal consequences of poorly controlled asthma. Discuss the importance of maintaining optimal asthma control throughout pregnancy	Airlyn^®^ was introduced as a supportive mHealth component, and participants were encouraged to become familiar with the application for subsequent breathing-exercise practice.	One individual educational session; 60–90 min	Review participants’ understanding through discussion and questions; reinforce key messages.
Week 3	Session 2: Asthma self-management, medication adherence, and self-monitoring	To promote appropriate asthma self-management, medication adherence, symptom recognition, and self-monitoring	Explain prescribed asthma medications and their role during pregnancy. Reinforce adherence to controller medication. Explain appropriate use of rescue medication. Discuss recognition of worsening asthma symptoms. Explain the importance of following the individualized asthma action plan. Demonstrate correct inhaler technique using placebo inhalers. Conduct return demonstration until correct technique is achieved.	Airlyn^®^ continued as a supportive tool within the overall self-management program.	One individual educational session; 60–90 min	Assess understanding through interactive discussion and return demonstration of inhaler technique.
Week 4	Session 3: Lifestyle modification, trigger avoidance, and rhythmic breathing exercises	To strengthen lifestyle practices, reduce exposure to asthma triggers, and develop appropriate breathing-exercise skills	Discuss avoidance of tobacco smoke, household dust, air pollution, strong odors, respiratory infections, cold air, allergens, and emotional stress. Discuss healthy diet, adequate hydration, sufficient sleep, appropriate physical activity, and stress management. Reinforce regular antenatal and asthma follow-up. Demonstrate rhythmic breathing exercises. Supervise participant practice and provide corrective feedback.	Demonstrate use of Airlyn^®^ for guided rhythmic breathing. Supervise participants while practicing the exercises using the application. Encourage independent home practice using Airlyn^®^.	One individual educational/practical session; 60–90 min. Home breathing practice 2–3 times daily.	Assess correct performance through demonstration and return demonstration; reinforce home practice instructions.
Week 5	Session 4: Reinforcement, emergency preparedness, and asthma self-management	To consolidate previously learned skills and improve preparedness for asthma deterioration and emergency situations	Review the main educational messages from previous sessions. Reinforce medication adherence and trigger avoidance. Reinforce continued breathing exercises. Discuss early warning signs of asthma deterioration. Explain situations requiring urgent medical attention. Discuss common difficulties in asthma management during pregnancy. Provide individualized counseling according to participants’ needs.	Reinforce continued use of Airlyn^®^ for breathing exercises. Address difficulties related to application use when identified.	One individual reinforcement session; 60–90 min	Interactive review, questions, individualized counseling, and assessment of participants’ ability to describe appropriate emergency actions.
Weeks 6–8	Follow-up and reinforcement phase	To reinforce knowledge and self-management behaviors, encourage adherence, and support continued breathing-exercise practice	Reinforce previously delivered educational messages. Encourage adherence to prescribed controller medication. Monitor reported asthma-related symptoms. Answer participants’ questions and provide individualized support. Review completion of intervention components. Encourage attendance at scheduled antenatal and asthma follow-up visits.	Encourage regular use of Airlyn^®^ for guided rhythmic breathing exercises. Address difficulties in using the application.	Weekly follow-up through telephone calls and WhatsApp messages. Breathing exercises continued 2–3 times daily at home.	Weekly telephone/WhatsApp follow-up; document participants’ questions, reported symptoms, adherence-related concerns, and need for reinforcement.
At the end of Week 8	post-intervention assessment	To evaluate changes following completion of the eight-week intervention	Conduct the post-intervention assessment.	Continued supportive use of Airlyn^®^ for breathing exercises	Final assessment at the end of Week 8	Re-administer the ACT and Asthma Adherence and Self-Monitoring Questionnaire. Maternal outcomes were subsequently assessed from medical/obstetric records following delivery, and neonatal outcomes were assessed from newborn medical records after delivery and before discharge.
Throughout Weeks 1–8	Intervention fidelity and standardization	To ensure consistency in intervention delivery and minimize variability in intervention exposure	All researchers followed the same predefined intervention protocol and educational sequence. Standardized PowerPoint presentations, educational brochures, verbal explanations, demonstrations, and return demonstrations were used. Attendance was documented for each session. Participants who missed a scheduled session received an individual make-up session covering the same content.	Airlyn^®^ was used consistently as a supportive educational tool for guided breathing exercises and did not replace nursing education.	Continuous throughout the intervention	Attendance and completion of intervention components were monitored throughout the eight-week period.

**Table 3:** Sociodemographic Characteristics of the Studied Groups (*N* = 140).

Characteristics	Study Group (*n* = 70)		Control Group (*n* = 70)		Chi-Square/Fisher’s Exact Test	
	NO	%	NO	%	X^2^	*p*
Age/years: 20 20–24 25–29 30–34 ≥35	5 15 20 18 12	7.1 21.4 28.6 25.7 17.2	6 14 22 15 13	8.6 20.0 31.4 21.4 18.6	0.533	0.970
Education: Read and write Primary Education Secondary Education University Education Postgraduate	5 8 25 25 7	7.2 11.4 35.7 35.7 10.0	6 12 24 23 5	8.6 17.1 34.3 32.9 7.1	1.328	0.857
Residence: Urban Rural	45 25	64.3 35.7	43 27	61.4 38.6	0.122	0.726
Occupation: Housewife Employee	40 30	57.1 42.9	42 28	60.0 40.0	0.118	0.731

Footnote: *p* > 0.05 indicates no statistically significant difference.

**Table 4:** Comparison of the Obstetric History among the Studied Group (*N* = 140).

Characteristics	Study Group (*n* = 70)		Control Group (*n* = 70)		Chi-Square/Fisher’s Exact Test	
	NO	%	NO	%	X^2^	*p*
Gravity: 1 2 3 ≥4	20 18 17 15	28.6 25.7 24.3 21.4	22 15 17 16	31.4 24.3 21.4 22.9	0.281	0.964
Parity:01 2 ≥3	18 20 17 15	25.7 28.6 24.3 21.4	20 18 17 15	28.6 25.7 24.3 21.4	0.211	0.976
History of Abortion: Yes No	48 22	68.6 31.4	50 20	71.4 28.6	0.136	0.712
Gestational Age: 12–16 weeks 17–20 weeks 21- 24 weeks 25- 28 weeks	18 22 17 13	25.7 31.4 24.3 18.6	14 33 13 10	20.0 47.1 18.6 14.3	3.625	0.305

Footnote: *p* > 0.05 indicates no statistically significant difference.

**Table 5:** Comparison of Asthma History among the Studied Group (*N* = 140).

Characteristics		Study Group (*n* = 70)		Control Group (*n* = 70)		Chi-Square/Fisher’s Exact Test	
		NO	%	NO	%	X^2^	*p*
Duration of asthma: <1 year 2–5 years 6–10 years >10 years	12 30 18 10		17.1 42.9 25.7 14.3	10 28 20 12	14.3 40.0 28.6 17.1	0.538	0.910
Asthma triggers: Allergens Air pollution/smoke exposure Strong odors/chemical irritants Respiratory infections Weather changes/cold air Physical exertion Emotional stress Contributing triggers	6 3 11 4 6 5 3 33		8.6 4.3 15.7 5.7 8.6 7.1 4.3 47.1	5 5 5 8 5 5 5 34	7.1 7.1 7.1 11.4 7.1 7.1 7.1 48.6	0.099 0.530 2.540 1.458 0.099 0.000 0.530 0.029	0.753 0.466 0.111 0.227 0.753 1.000 0.466 0.866
History of Asthma Hospitalization: No Yes	40 30		57.1 42.9	38 32	54.3 45.7	0.116	0.734
History of Emergency Visits Yes No	35 35		50.0 50.0	36 34	51.4 48.6	0.029	0.866
Adherence to Asthma Treatment: Regular use Irregular use	30 40		42.9 57.1	28 42	40.0 60.0	0.118	0.731
Asthma Severity: Mild Moderate Severe	30 25 15		42.9 35.7 21.4	28 26 16	40.0 37.1 22.9	0.121	0.941

Footnote: *p* > 0.05 indicates no statistically significant difference. For Triggers and Medications, women could report more than one item; percentages are calculated out of the group total and do not sum to 100%.

**Table 6:** Comparison of the Asthma Control Test (ACT) Total Scores and Asthma Control Level between the Studied Groups before and after the Intervention.

Total Asthma Control Test	Pre–Intervention					Post–Intervention				
	Study Group (*n* = 70)		Control Group (*n* = 70)		Sig. Test	Study Group (*n* = 70)		Control Group (*n* = 70)		Sig. Test
	NO	%	NO	%		NO	%	NO	%	
Uncontrolled asthma	62	88.6	61	87.1	X^2^ = 0.067, *p* = 0.769	17	24.3	58	82.9	X^2^ = 48.275, *p* < 0.001 **
Well-controlled asthma	8	11.4	9	12.9		53	75.7	12	17.1	
Mean ± SD	14.5 ± 4.2		14.1 ± 4.3		t = 0.555, *p* = 0.580	19.7 ± 3.6		15.2 ± 4.2		t = 6.789, *p* < 0.001 **
Effect size (Cohen’s d)	0.094					1.147				
95% CI	[−1.026, 1.826]					[3.189, 5.811]				

Footnote: ** *p* < 0.001 indicates a highly statistically significant difference. ACT score interpretation: ≤19 = uncontrolled asthma; 20–25 = well-controlled asthma.

**Table 7:** Comparison of the Asthma Adherence and Self-Monitoring overall score and level between the Studied Groups before and after the Intervention.

Total Asthma Adherence and Self-Monitoring	Pre–Intervention					Post–Intervention				
	Study Group (*n* = 70)		Control Group (*n* = 70)		Sig. Test	Study Group (*n* = 70)		Control Group (*n* = 70)		Sig. Test
	NO	%	NO	%		NO	%	NO	%	
Well asthma adherence	7	10.0	9	12.9	X^2^ = 1.835, *p* = 0.400	50	71.4	11	15.7	X^2^ = 45.580, *p* < 0.001 **
Moderate asthma adherence	22	31.4	28	40.0		13	18.6	28	40.0	
Poor asthma adherence	41	58.6	33	47.1		7	10.0	31	44.3	
Mean ± SD	10.1 ± 2.7		9.3 ± 3.2		T = 1.591, *p* = 0.114	5.6 ± 2.7		9.1 ± 3.1		T = 7.092, *p* < 0.001 **
Effect size (Cohen’s d)	0.269					1.199				
95% CI	[−0.194, 1.794]					[−4.476, −2.524]				

Footnote: ** *p* < 0.001 indicates a highly statistically significant difference.

**Table 8:** Comparison of Maternal Outcomes between the Studied Groups after the Intervention.

Items	Study Group (*n* = 70)		Control Group (*n* = 70)		Chi-Square/Fisher’s Exact Test	
	NO	%	NO	%	X^2^	*p*
Gestational Hypertensive disorders	26	37.1	40	57.1	5.618	0.018 *
Gestational diabetes mellitus	22	31.4	35	50.0	5.001	0.025 *
Respiratory infections during pregnancy	30	42.9	41	58.6	3.458	0.063
Urinary tract infection (UTI)	24	34.3	45	64.3	12.603	<0.001 **
Preterm labor or PPROM	26	37.1	43	61.4	8.259	0.004 *
Antepartum hemorrhage	27	38.6	43	61.4	7.314	0.007 *
Postpartum hemorrhage	25	35.7	44	62.9	10.316	<0.001 **
Others (Venous Thromboembolism)	29	41.4	45	64.3	7.338	0.007 *
Mode of Delivery						
Vaginal delivery	43	61.4	17	24.3	30.972	<0.001 **
Elective cesarean section	17	24.3	12	17.1		
Emergency cesarean section	10	14.3	41	58.6		

Footnote: * *p* < 0.05 indicates a statistically significant difference; ** *p* < 0.001 indicates a highly statistically significant difference.

**Table 9:** Comparison of Total Maternal Outcome Score and Level between the Studied Groups after the Intervention.

Total Maternal Outcome	Study Group (*n* = 70)		Control Group (*n* = 70)		Sig. Test
	NO	%	NO	%	
Good outcome	51	72.9	12	17.1	X^2^ = 44.840, *p* < 0.001 **
Moderate outcome	12	17.1	28	40.0	
Poor outcome	7	10.0	30	42.9	
Mean ± SD	3.5 ± 1.7		6.1 ± 2.6		T = 6.947, *p* < 0.001 **
Effect size (Cohen’s d)	1.174				
95% CI	[−3.340, −1.860]				

Footnote: ** *p* < 0.001 indicates a highly statistically significant difference.

**Table 10:** Comparison of Neonatal Outcomes between the Studied Groups after the Intervention.

Items	Study Group (*n* = 70)		Control Group (*n* = 70)		Chi-Square/Fisher’s Exact Test	
	NO	%	NO	%	X^2^	*p*
Birth weight						
Very low birth weight (<1500 g)	6	8.6	20	28.6	9.518	0.009 *
Low birth weight (<2500 g)	14	20.0	13	18.6		
Normal (≥2500 g)	50	71.4	37	52.9		
Apgar score at 1 min						
0–3 (severe depression)	10	14.3	26	37.1	9.833	0.007 *
4–6 (moderate depression)	15	21.4	13	18.6		
7–10 (normal)	45	64.3	31	44.3		
Apgar score at 5 min						
0–3 (severe depression)	8	11.4	27	38.6	18.905	<0.001 **
4–6 (moderate depression)	13	18.6	18	25.7		
7–10 (normal)	49	70.0	25	35.7		
Respiratory distress after birth						
No	53	75.7	36	51.4	8.914	0.003 *
Yes	17	24.3	34	48.6		
Type of respiratory support required (if applicable)						
Oxygen therapy only	5	29.4	20	58.8	5.571	0.062
CPAP	8	47.1	6	17.6		
Mechanical ventilation	4	23.5	8	23.5		
NICU admission						
No	53	75.7	34	48.6	10.961	<0.001 **
Yes	17	24.3	36	51.4		
Duration of NICU stay (if admitted)						
<24 h	2	11.8	15	41.7	5.034	0.081
1–3 days	8	47.1	13	36.1		
>3 days	7	41.2	8	22.2		
Neonatal outcome status at discharge						
Stable/healthy	43	61.4	33	47.1	5.161	0.075
Complications requiring follow-up	19	27.1	19	27.1		
Critical condition	8	11.4	18	25.7		

Footnote: * *p* < 0.05 indicates a statistically significant difference; ** *p* < 0.001 indicates a highly statistically significant difference.

**Table 11:** Comparison of Total Neonatal Outcomes Score and Level between the Studied Groups after the Intervention.

Total Neonatal Outcome	Study Group (*n* = 70)		Control Group (*n* = 70)		Sig. Test
	NO	%	NO	%	
Poor neonatal outcome	3	4.3	10	14.3	X^2^ = 31.290, *p* < 0.001 **
Moderate neonatal outcome	17	24.3	43	61.4	
Good neonatal outcome	50	71.4	17	24.3	
Mean ± SD	7.7 ± 2.2		5.5 ± 2.1		T = 6.047, *p* < 0.001 **
Effect size (Cohen’s d)	1.022				
95% CI	[1.480, 2.920]				

Footnote: ** *p* < 0.001 indicates a highly statistically significant difference.

## Data Availability

The data presented in this study are available from the corresponding authors upon reasonable request due to privacy and ethical restrictions.

## Supplementary Materials

The following supporting information can be downloaded at https://www.mdpi.com/article/10.3390/healthcare14183079/s1, Table S1: Comparative Summary: Recent Literature vs. Present Study.
