# Supplementary material for: Effect of Nursing Intervention Supported by Mobile Health Applications on Asthma Control, Maternal and Neonatal Outcomes Among Pregnant Women with Asthma
Source: Healthcare (Basel). 2026 Sep 19;14(18):3079. doi: 10.3390/healthcare14183079 (PMC13606770; doi:10.3390/healthcare14183079)
Supplement: Supplementary file 1 [file healthcare-14-03079-s001.zip › healthcare-4478006-supplementary.pdf]

Table S1. Comparative Summary: Recent Literature vs. Present Study

Reference & Year	Study Population & Design	Intervention & mHealth Type	Outcome Measures & Main Findings	Key Limitations	Present Study Comparison / Findings
Present Study (2026)	• Pop: Pregnant women with asthma<br>• Design: Quasi-experimental	• Intervention: Nursing intervention (education, inhaler technique, reminders)<br>• mHealth: Mobile health apps	• Measures: ACT, adherence, self-monitoring, maternal & neonatal outcomes<br>• Findings: Significant improvement in ACT, adherence, self-monitoring; reduced maternal/neonatal complications	Non-randomized design; cannot definitively establish causality; underlying mechanisms uncertain	Baseline: High rate of uncontrolled asthma & non-adherence across groups.<br>Post-Intervention: majority of intervention group achieved well-controlled asthma, higher adherence, and significantly better outcomes.
Er et al. [35] (2026)	• Pop: Pregnant women / Maternal health cohort<br>• Design: Systematic review	• Intervention: mHealth with behavior change techniques<br>• mHealth: Apps, web platforms, smart bands, SMS	• Measures: Maternal health outcomes<br>• Findings: Significant positive effects of mHealth tools on maternal outcomes.	Varied behavior change techniques across included studies	Supports present study's conclusion that mHealth-supported nursing interventions significantly enhance maternal well-being.
Mohamed et al. [10, 33] (2025)	• Pop: Asthmatic patients<br>• Design: Experimental / Interventional	• Intervention: App-based education & follow-up<br>• mHealth: Smartphone application	• Measures: ACT score, adherence, QoL, exacerbations<br>• Findings: Higher mean ACT, adherence, QoL; significantly lower exacerbations & hospitalizations.	Specific to local population (Upper Egypt)	Supports present findings on mHealth efficacy in improving ACT scores, treatment adherence, and clinical outcomes.
Thomas et al. [26] (2025)	• Pop: Pregnant women with asthma<br>• Design: Randomised controlled tele-trial	• Intervention: Virtual antenatal asthma care & self-management<br>• mHealth: Telehealth / Virtual platform	• Measures: ACQ score, proportion well-controlled<br>• Findings: Significant improvement in ACQ score; larger proportion achieved well-controlled asthma status.	Did not isolate relative contribution of individual intervention components	Parallels present study's finding of improved asthma control via digital/virtual support platforms during pregnancy.

Reference & Year	Study Population & Design	Intervention & mHealth Type	Outcome Measures & Main Findings	Key Limitations	Present Study Comparison / Findings
Sebhi et al. [27] (2025)	• Pop: Asthmatic patients<br>• Design: 6-month educational follow-up	• Intervention: Therapeutic education program<br>• mHealth: N/A (Clinical/Educational)	• Measures: Knowledge, adherence, control, lung function<br>• Findings: Significant improvements in adherence and self-monitoring post-intervention.	Non-pregnant adult cohort focus	Directly mirrors present study's outcome of enhanced treatment adherence and self-monitoring post-nursing intervention.
Gebresilassie et al. [11] (2025)	• Pop: Asthmatic patients<br>• Design: Quasi-experimental study	• Intervention: Asthma education intervention<br>• mHealth: N/A (Standard education)	• Measures: Knowledge, asthma control level<br>• Findings: Significantly higher asthma control scores in intervention group post-intervention.	Non-randomized design	Consistent baseline uncontrolled asthma; aligns with significant post-intervention ACT score improvements.
Ameyaw et al. [34] (2024)	• Pop: Maternal health cohorts<br>• Design: Systematic review of reviews	• Intervention: mHealth delivery interventions<br>• mHealth: Mobile apps & SMS messaging	• Measures: Maternal healthcare use, anxiety, weight<br>• Findings: mHealth apps effectively improved maternal healthcare outcomes and behavior.	Heterogeneity across systematic reviews	Corroborates positive role of mHealth platforms on maternal behavioral modification and healthcare utilization.
Georgakopoulou et al. [29] (2024)	• Pop: Pregnant women with asthma<br>• Design: Narrative Review	• Intervention: Asthma treatment & management behaviors<br>• mHealth: N/A	• Measures: Maternal-fetal outcomes, adverse risks<br>• Findings: Lower risks of adverse neonatal outcomes and positive association with pregnancy outcomes.	Narrative review limitations	Reinforces positive correlations found in present study between management behaviors and maternal/fetal health.

Reference & Year	Study Population & Design	Intervention & mHealth Type	Outcome Measures & Main Findings	Key Limitations	Present Study Comparison / Findings
Haastrup et al. [28] (2026)	• Pop: Pregnant women with asthma<br>• Design: Systematic review	• Intervention: Asthma management vs. usual care<br>• mHealth: N/A	• Measures: Perinatal outcomes, maternal risk correlation<br>• Findings: Majority of intervention group achieved good perinatal outcomes compared to usual care.	Observational /review study bounds	Matches present study's findings on higher rate of favorable maternal/perinatal outcomes and inverse ACT-adverse outcome correlation.
